# Global Identification and Characterization of C2 Domain-Containing Proteins Associated with Abiotic Stress Response in Rice (*Oryza sativa* L.)

**DOI:** 10.3390/ijms23042221

**Published:** 2022-02-17

**Authors:** Hongjia Zhang, Yuting Zeng, Jeonghwan Seo, Yu-Jin Kim, Sun Tae Kim, Soon-Wook Kwon

**Affiliations:** 1 Department of Plant Bioscience, College of Natural Resources and Life Science, Pusan National University, Miryang 50463, Korea; hjzhangedu@outlook.com (H.Z.); zyt20210921@163.com (Y.Z.); rightseo@hotmail.com (J.S.); stkim71@pusan.ac.kr (S.T.K.); 2 Life and Industry Convergence Research Institute, Pusan National University, Miryang 50463, Korea; 3 Department of Life Science & Environmental Biochemistry, College of Natural Resources and Life Science, Pusan National University, Miryang 50463, Korea; yjkim2020@pusan.ac.kr

**Keywords:** rice, C2 domain-containing protein (OsC2DP), abiotic stress, haplotype, phylogenetics, expression pattern

## Abstract

C2 domain-containing proteins (C2DPs) have been identified in different genomes that contain single or multiple C2 domains in their C- or N-terminal. It possesses higher functional activity in the transmembrane regions. The identification of C2 domains were reported in a previous study, such as multiple C2 domains and transmembrane-region proteins (MCTPs) and N-terminal-TM-C2 domain proteins (NTMC2s) of rice, *Arabidopsis thaliana*, and cotton, whereas the *C2DP* gene family in rice has not been comprehensively studied, and the role of the *C2DP* gene in rice in response to abiotic stress is not yet fully understood. In this study, we identified 82 C2DPs in the rice genome and divided them into seven groups through phylogenetic analysis. The synteny analysis revealed that duplication events were either exhibited within the genome of rice or between the genomes of rice and other species. Through the analysis of *cis*-acting elements in promoters, expression profiles, and qRT-PCR results, the functions of *OsC2DPs* were found to be widely distributed in diverse tissues and were extensively involved in phytohormones-related and abiotic stresses response in rice. The prediction of the microRNA (miRNA) targets of *OsC2DPs* revealed the possibility of regulation by consistent miRNAs. Notably, *OsC2DP50/51/52* as a co-tandem duplication exhibited similar expression variations and involved the coincident miRNA-regulation pathway. Moreover, the results of the genotypic variation and haplotype analysis revealed that *OsC2DP17*, *OsC2DP29*, and *OsC2DP49* were associated with cold stress responses. These findings provided comprehensive insights for characterizations of *OsC2DPs* in rice as well as for their roles for abiotic stress.

## 1. Introduction

Calcium (Ca) is a necessary nutrient ingredient for plant development as it serves as a signaling factor for transduction functions in multiple physiological processes [1]. Ca maintains the steadiness of the cell wall, membrane, and membrane-binding proteins and improves the resistance to diverse abiotic and biotic stresses in plant cells, which thereby trigger multiple signaling pathways [2,3]. Ca-binding protein is a kind of protein for specific binding to the intracellular free Ca^2+^, the signaling transduction function created by binding to each other. To date, three major Ca-binding proteins, namely calmodulins [4], Ca-dependent protein kinase [5], and calcineurin B-like proteins [6], have been identified as well-known Ca^2+^ sensors.

C2DPs, a type of Ca-dependent protein kinase, are highly conserved in long term evolutionary process. The typical C2 domain consists of approximately 135 amino acids and was given the name PKC-C2 (PFAM: PF00168) in a previous study [7]. The other two branches of the C2 domain super family include the PI3K-C2 (PFAM: PF00792) and PTEN-C2 domains (PF10409). Most PKC-C2 proteins are involved in Ca^2+^-dependent interactions with the membrane, and most previous reports have characterized PKC-C2 [8]. Generally, C2DPs contain at least one C2 domain, with or without transmembrane domains in the C-terminus of the protein. However, several C2 domains are frequently coupled to other enzymatic domains, such as the phospholipase domain [9,10], synaptotagmin domain [11], and phosphatidylinositol domain [12], which bind to the membrane or point to other functions. With the development of the protein structure, a set of multiple C2 domains and transmembrane region proteins were identified as branches of C2DPs [13], which contained between three and four C2 domains at the N-terminus and between one and four transmembrane regions at the C terminus. In membrane transport proteins, multiple C2 domains have been found to have different but conserved sequences, suggesting the formation of more biological functions due to the cooperation or crosstalk between multiple C2 domains [14].

Related studies concerning C2 domains have been published related to eukaryons. The first characterized C2 domain was found in mammalian cells, and the animal PKC contained from three to four conserved domains named C1, C2, C3, and C4. Interestingly, C1, C3, and C4 exist in all PKCs; the C2 domain only exists in Ca^2+^-dependent PKC, indicating a synergistic relationship between the Ca^2+^ and C2 domains [15]. The function of the C2 domain has also been studied [16,17]. The C2 domain mediates the transduction mechanism whereby peptides derived from PKC modulate protein–protein interactions within PKC and the PKC binding proteins [18]. The peptides derived from the C2 domain were also identified as two branches, the isozyme-selective activator and the selective inhibitor of epsilon PKC, which play important roles in the prevention and protection of cardiac and brain ischemic damage, diabetes complications, and transplanted hearts [19].

The functions of C2DPs have also been reported in plants within the last 20 years [20,21,22]. In *Arabidopsis thaliana*, gene *SYT1*, which belongs to synaptotagmins, was identified by the affected viability of cells as a consequence of a decrease in the integrity of the plasma membrane [11]. The *QKY* gene has four predicted C2 domains and was the first characterized MCTP in *Arabidopsis thaliana*. *Qky* mutant plants that showed defects in the development of the outer integument, the floral organ, and the stem and impacted the growth of the floral meristem and the root hair [23]. In rice, *OsERG1a* and *OsERG1b*, which are the first reported C2DP, expression of *OsERG1* increased by fungal elicitor of *Magnaporthe grisea* and calcium ionophore (10 mM of Ca^2+^ and 20 µM of A23187), the subcellular localization of OsERG1 could be from the cytoplasm to the cell membrane, and participates in the plant defense signaling pathway [24]. OsSMCP1, a Ca^2+^-dependent membrane-targeting domain protein, contains a single C2 domain, and the overexpression of OsSMCP1 enhances a plant’s tolerance to salt, drought, and osmotic as well as oxidative stress, and improves its resistance to *Pseudomonas syringae* [25]. OsC2DP (LOC_Os09g39770) is also significantly involved in salt tolerance in rice. The *OsC2DP* CRISPR/Cas9 knockout mutant line was found to be more sensitive to salt stress than wild type (WT), and salt treatment caused the translocation of OsC2DP from the cytoplasm to the cell membrane [26], which was similar to the shift identified for the protein, OsERG1 [24]. To date, the research progress of the C2 domain has been reported in only a few species, such as in *Arabidopsis thaliana* and cotton, where studies revealed the results for MCTPs, a branch of the C2DP family that was divided into five subfamilies according to the phylogenetic trees [27,28]. In rice, a comprehensive analysis of NTMC2s revealed 13 family members divided into six subfamilies according to phylogeny and motif constitutions [29]. Therefore, the *C2DP* gene family of rice requires further comprehensive analysis to identify the relationships and functions of the members.

In this study, 82 family members of the C2DP family were identified in rice and named the *OsC2DP* gene family. In addition, seven subgroups were identified based on their homologous relationships and their conserved domain structures. Our results provide a theoretical basis for the future characterization of the roles of OsC2DPs in abiotic stress in rice.

## 2. Results

### 2.1. Identification, Phylogenetic, and Sequence Analyses of OsC2DPs

To identify the *OsC2DP* gene family members in rice, a hidden Markov model (HMM) search was performed in a rice proteome database using the HMM model (PF00168). As a result, 82 OsC2DPs were identified by filtering an E-value < 1 × 10^−5^ (Supplementary Table S1), and the 82 candidates’ sequences were subsequently submitted to the SMART and InterPro web tools to verify the C2 domain. The whole OsC2DPs were found to have different basic characteristics (Supplementary Table S2). Furthermore, the genome DNA length ranged from 784 (OsC2DP67) to 13,170 bp (OsC2DP62), with an average of 4484 bp, while the cds length ranged from 432 (OsC2DP20) to 6354 bp (OsC2DP80), with an average of 1875 bp. The physical properties were also revealed to be widespread (Supplementary Table S2). The pI of OsC2DPs ranged from 4.22 (OsC2DP36) to 11.23 (OsC2DP12), with an average of 7.13; 46 members had a pI < 6 while 31 members had a pI > 8. The *M*_W_ of OsC2DPs ranged from 8.847 (OsC2DP54) to 227.943 kD (OsC2DP80), with an average of 65.62 kD. The chromosomal distribution results showed that OsC2DPs were present in each chromosome (Supplementary Figure S1), except in chr08, chr10, chr11, and chr12. There have been many family members that exist in other chromosomes. Interestingly, there were some gene clusters in small, specific regions, such as OsC2DP7 and OsC2DP8 in chr01 as well as OsC2DP67 and OsC2DP68 in chr07. A larger cluster was identified in chr06, which included OsC2DP50–OsC2DP57, implying that unknown tandem or segmental duplications might exist in a few regions.

**Figure 1:**
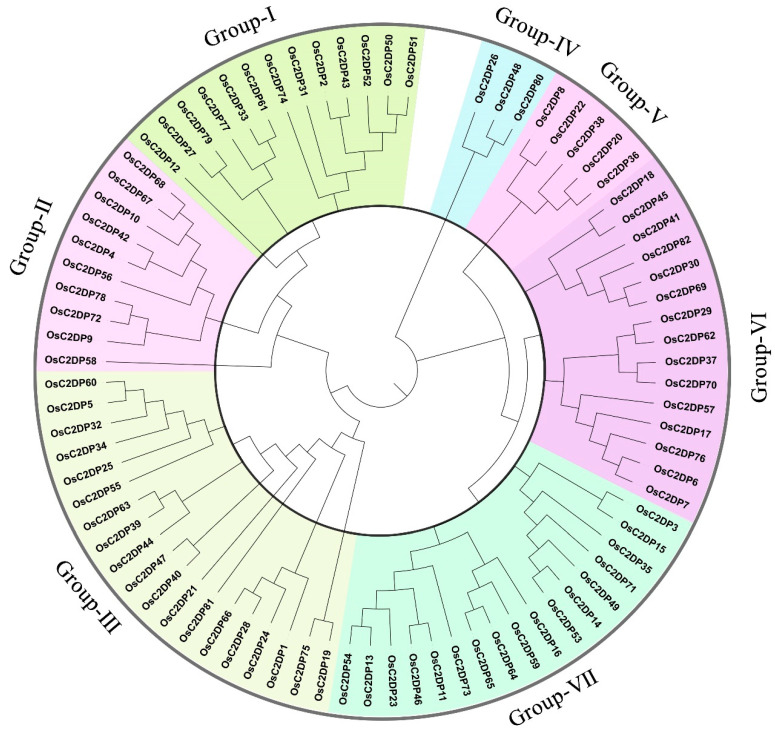
Phylogenetic tree of OsC2DPs in rice genome. Diverse colors represent different groups, Neighbor-joining (NJ) function was performed by the substitution model of p-distance for amino acid and phylogeny test of 1000 times Bootstrap replications.

**Figure 2:**
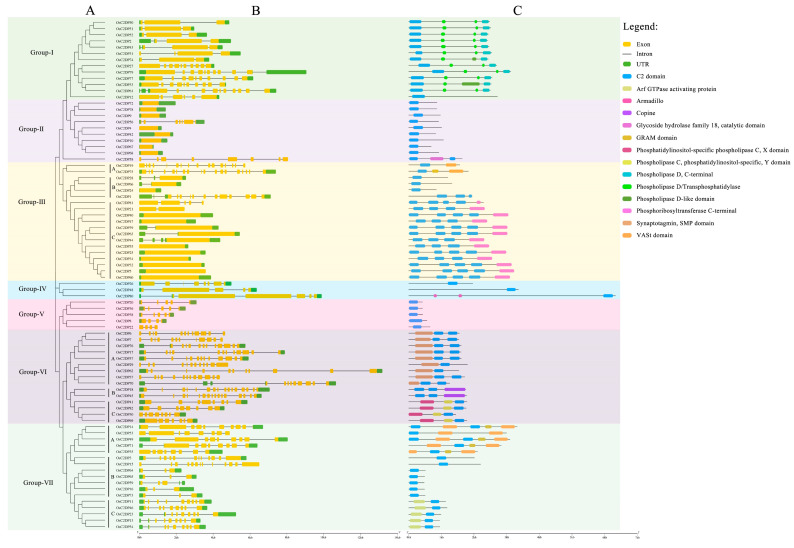
Structural schematic diagram of OsC2DP gene family. (**A**) Phylogenetic relationship of OsC2DPs. A, B, and C represent different class of each group. (**B**) Gene structure of OsC2DPs. Green color, yellow color, and solid line represent exon, UTR, and intron region. (**C**) Putative domain structure of OsC2DPs. Diverse colors represent domains followed by the legend of figure.

To further detect the diverse functions of OsC2DPs, the phylogenetic relationships and the gene and domain structures were analyzed (Figure 1 and Figure 2). The neighbor-joining (NJ) tree showed that 82 members was divided into seven groups, among which, most (i.e., 19 members) gathered in Group-III and the least (i.e., 3 members) gathered in Group-IV (Figure 1). In the structure analysis, the C2 domains were consistently identified, and single or multiple C2 domains were consistently present in each family member. The members of the seven groups had diverse structures, and the structures effectively supported the grouping results of the phylogenetic trees (Figure 2). In Group-I, 12 out of the 13 members contained 2 phospholipase D/transphosphatidylase domains and a single phospholipase D’s C-terminal domain. Only OsC2DP12 contained other types and had distant relationships with other group members. In addition, the results of multiple sequence alignments showed that this domain was highly conserved among the members (Supplementary Figure S2). Group-II and Group-V showed similar domain structures (only a single C2 domain was found). However, most members of Group-II had a simple gene structure that contained one exon; Group-V members showed opposite results with multiple exon regions. Group-III had 19 members with one cluster of a phylogenetic tree, based on the mixture structures, and we further divided them into three classes, A, B, and C; Class C is a special class that showed a simple gene structure and a phosphoribosyltransferase C-terminal. The results of multiple sequence alignments revealed a high homology between members of this class (Supplementary Figure S3). In Group-VI and Group-VII, similar groupings were displayed and divided into the three classes, A, B, and C, according to their diverse domain structures. In Group-VI Class A and Class C, there were single and two highly conserved domains, respectively. Synaptotagmin, SMP domain in Class A (Supplementary Figure S4); phosphatidylinositol-specific phospholipase C, X domain; and phospholipase C, phosphatidylinositol-specific, Y domain in Class C (Supplementary Figure S5).

These results suggested that OsC2DPs is a vast gene family and performs diverse roles in plants. Members with a close relationship might possess similar functions, and diverse domains also involve differentiation in each group. Notably, the most conserved phospholipase-related domains were found in OsC2DPs, implying that they might be involved in abiotic stress, ionic transport, or exchange functions.

### 2.2. Synteny Analysis and Ka/Ks Ratio of OsC2DPs in Rice and Others Genome

**Figure 3:**
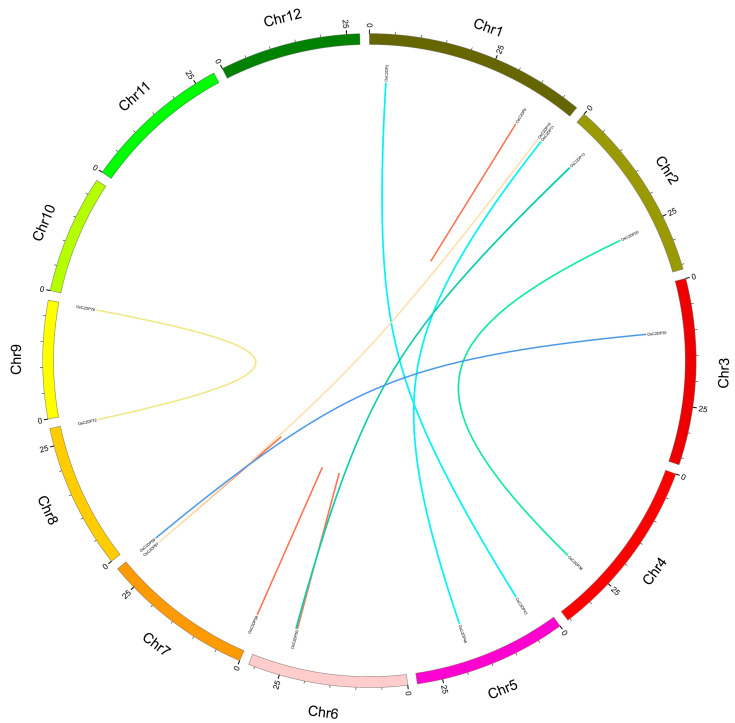
Synteny analysis of OsC2DPs intra rice genome. Each rice chromosome is displayed in different color. Duplicated gene pairs are displayed and linked using lines with the color, red half lines represent tandem duplications.

The analysis of tandem or segmental duplications could explain the derivation of gene family duplication events. According to a previous study, alignment ratios greater than 70% were identified as duplications while gene pairs within the closed region (100 kb) were selected as tandem duplications. The results are shown in Figure 3, where the red and other colored lines represent tandem and segmental duplications, respectively. Six tandem duplications (OsC2DP6/7, OsC2DP50/51, OsC2DP51/52, OsC2DP50/52, OsC2DP58/59, and OsC2DP67/68) and eight segmental duplications (OsC2DP11/46, OsC2DP2/43, OsC2DP10/67, OsC2DP20/36, OsC2DP13/54, OsC2DP14/53, OsC2DP30/69, and OsC2DP72/78) were found in the rice genome. Interestingly, three genes, OsC2DP50, OsC2DP51, and OsC2DP52, were involved in a co-duplication event in a narrow region (19.784 kb), implying that these genes might have the same function or signaling pathway, or possess a redundant effect in the regulation process. We also analyzed the evolutionary relationships and orthologous genes of C2DPs between rice and other popular crop genomes. A total of 96, 100, 93, 82, 187, and 302 C2DPs were identified in *Arabidopsis thaliana*, barley, maize, sorghum, soybean, and wheat genomes, respectively; the results are shown in Supplementary Figure S6. Among these, *Arabidopsis thaliana* and wheat showed the minimum and maximum numbers, with 8 and 176 orthologous gene pairs, respectively (Supplementary Figure S6A,F). A total of 53, 83, 74, and 26 orthologous gene pairs were found between rice and barley, maize, sorghum, and soybean, respectively (Supplementary Figure S6B–E). Such findings indicate that *Arabidopsis thaliana* showed the most distant relationship with rice while wheat showed the closest, and the C2DP gene family has been conserved differently among diverse species, possibly to maintain a particular biological function.

In genetic studies, the Ka/Ks ratio represents the comparison of the non-synonymous substitution rate (Ka) and the synonymous substitution rate (Ks) of duplications. Therefore, the Ka/Ks ratio could determine whether there was selective pressure for this gene pair [30]. In previous results, we identified duplications and orthologous gene pairs in rice and other genomes; therefore, we calculated the Ka/Ks ratio for understanding the evolutionary model of OsC2DPs. All Ka/Ks ratios of the tandem and segmental duplications were less than one (Table 1), suggesting that these duplications involved purified (negative) selection. The calculated results of *Arabidopsis thaliana*, barley, maize, sorghum, soybean, and wheat were similar to those of rice, with Ka/Ks ratios less than one (Supplementary Tables S3–S8), and involved purified (negative) selection. Notably, there was one orthologous gene pair (OsC2DP58/59) with a Ka/Ks ratio of 0.905 (Table 1), which was as close to 1 as the neutral evolution. Another gene pair (OsC2DP67-TraesCS2A02G115700) had a Ka/Ks ratio of 1.069 in the rice–wheat orthologous pair (Supplementary Table S8), suggesting that the orthologs involved positive selection.

### 2.3. Expression Profile Analysis of OsC2DPs in Rice Growth Stage

**Figure 4:**
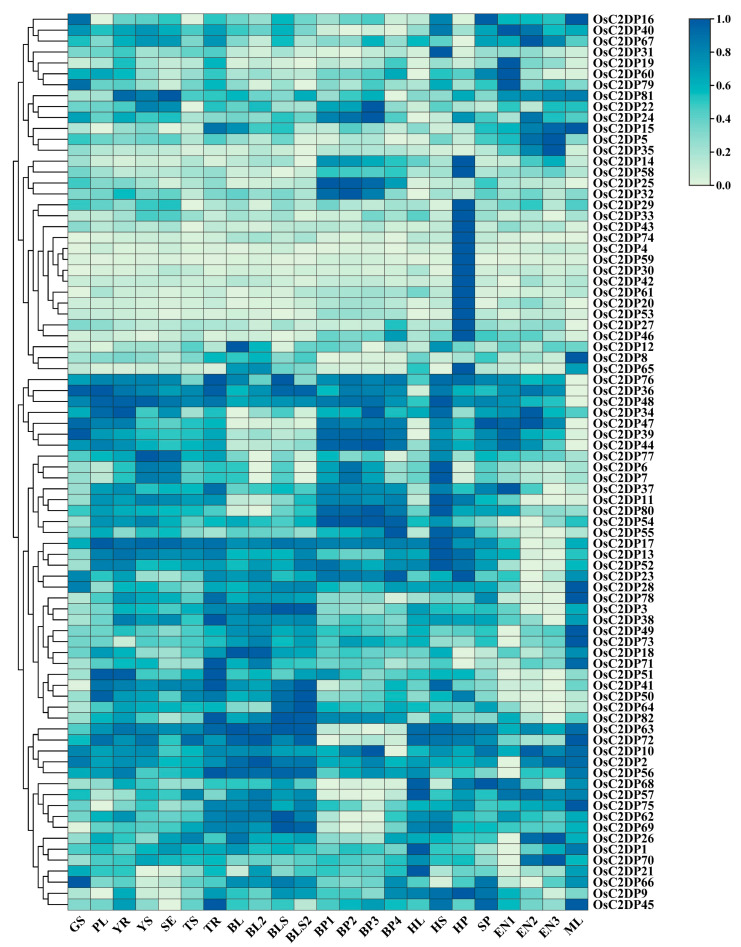
Expression profiles of OsC2DPs in diverse tissues across different stages. Data collected from expression database NCBI GEO, accession ID: GSE19024, data processing followed averaged by three times repeats, log 10-transformed, row normalization, and cluster. GS: germinating seed; PL: plumule; YR: radicle; YS: young seedling; SE: seedling at trefoil stage; TS: shoot under 2 tillers; TR: root under 2 tillers; BL: mature leaf blade under young panicle; BL2: mature leaf blade under mature panicle; BLS: mature leaf sheath under young panicle; BLS2: mature leaf sheath under mature panicle; BP1: developing panicle (length < 1 mm); BP2: developing panicle (3 < length < 5 mm); BP3: developing panicle (10 < length < 15 mm); BP4: developing panicle (40 < length < 50 mm); HL: flag leaf in heading date; HS: stem in heading stage; HP: panicle in heading stage; SP: spikelet; EN1: endosperm under 7 days after pollination; EN2: endosperm under 14 days after pollination; EN3: endosperm under 21 days after pollination; ML: flag leaf under mature stage.

The change in the transcriptional level of genes could explain the vitality of a gene in diverse tissues and growth stages. To determine the specific function of the gene at spatiotemporal variations, we analyzed the transcriptome data for the detected expressions of OsC2DPs during different tissues and stages in rice. Diverse expression patterns were observed in the OsC2DPs (Figure 4). For example, OsC2DP16, OsC2DP40, OsC2DP67, and OsC2DP81, among others, showed higher expression in endosperm during the 7–21 days post-pollination stage (EN 1–3) and flag leaf during the mature stage (ML), suggesting that these genes function in later, mature stages. OsC2DP29, OsC2DP33, OsC2DP43, OsC2DP74, OsC2DP4, OsC2DP59, and OsC2DP30, among others, showed the highest expression in the panicle during the heading stage (HP), suggesting that these genes may perform major functions in the panicle, specifically. Additionally, genes such as OsC2DP47, OsC2DP39, and OsC2DP44 or OsC2DP37, OsC2DP11, OsC2DP80, and OsC2DP54, had higher expression in the panicle during the developing stage (BP 1–4) while OsC2DP51, OsC2DP41, OsC2DP50, and OsC2DP64, among others, had lower expression in the endosperm (Figure 4). These diverse expression patterns indicated that the group clusters enabled some genes to perform certain functions by being clustered with other genes. Based on this conclusion, we further analyzed the expression profiles of the tandem and segmental duplications. OsC2DP50/51/52 were three genes showing co-tandem duplications that contained similar expression variations across the entire growth stage in rice (Supplementary Figure S7A). OsC2DP58 and OsC2DP59 showed differences at an earlier stage but maintained consistent expression variations in flag leaf during the heading date (HL) and spikelet phase (SP) (Supplementary Figure S7B). Further, there were no significant similarities in phenomena in OsC2DP67/68 (Supplementary Figure S7C). In segmental duplications, there were two gene pairs; the two gene pairs showed completely similar and partially similar expression variations, respectively (Supplementary Figure S8). Among these, OsC2DP14/53 and OsC2DP72/78 showed similar expression variations across the whole growth stage (Supplementary Figure S8D,H). OsC2DP11/46 was similar in the heading stage and endosperm (Supplementary Figure S8A) while OsC2DP13/54 showed similar variations from germination to tiller and mature stages (Supplementary Figure S8B).

Taken together, OsC2DPs were found to be widely expressed in whole tissues and growth stages. Further, a diverse expression pattern was observed in the gene cluster, suggesting that the duplications were likely involved in the same functions and regulation pathways in specific tissues and stages via their collaborative expression variations.

### 2.4. Prediction and Analysis of the Cis-Acting Elements in Promoter Regions of OsC2DPs

Generally, the gene response to treatments or other functions would be achieved by changing the activity of the treatment-related *cis*-acting elements [31]. In this study, we analyzed the abiotic stress- and phytohormone-related *cis*-acting elements in the promoter region of the OsC2PDs. Results were validated functions of the OsC2DP gene family were related to abiotic stress and phytohormone management, shown in Supplementary Figure S9 and more detailed information in Supplementary Table S9. A total of seven types of abiotic-stress-responsive elements and five types of phytohormone-responsive elements were identified: the cold-responsive element, LTR (CCGAAA); the drought-responsive element, MBS (CAACTG); the salt-responsive element, GT1GMSCAM4 (GAAAAA); the heat-responsive element, CCAATBOX1 (CCAAT); the diverse-light-responsive elements, ACE (GACACGTATG), G-box (TACGTG), GT1-motif (GGTTAA), etc.; the circadian element, CAAAGATATC; and the wound-responsive element, WUN-motif (AAATTTCCT). Phytohormone-responsive elements including abscisic acid (ABA) (ABRE: ACGTG/CACGTG), auxin (IAA) (TGA-element: AACGAC and AuxRR-core: GGTCCAT), gibberellin (GA) (TATC-box: TATCCCA and GARE-motif: TCTGTTG), salicylic (SA) (TCA-element: CCATCTTTTT), and jasmonic (JA) (TGACG-motif: TGACG and CGTCA-motif: CGTCA) were found. After statistical analysis, a total of 923 light-responsive elements and 390 JA-responsive elements were found as the most abundant *cis*-acting elements in OsC2DPs. For each gene, the light-responsive element most enriched for OsC2DP5 (28), OsC2DP29 (22), OsC2DP3 (20), and OsC2DP64 (20), implied that the expressions of these gene functions were possibly involved in the light-signaling pathway in rice. In addition, some genes contained most of the elements with multiple functions; for example, OsC2DP5 contained most of the elements related to salt and light; OsC2DP29 contained most of the elements related to cold, salt, and light; and OsC2DP28 contained most of the elements related to drought and heat. These results indicated that the OsC2DP gene family is possibly widely involved in abiotic and phytohormone response in rice.

### 2.5. Expression Analysis of OsC2DPs Involved Abiotic and Exogenous Phytohormone Treatments

To further verify the function of genes in abiotic stress response and whether it could respond to exogenous plant hormone-type compounds, we collected transcriptome data and performed qRT-PCR to detect the variations in the transcriptional levels in the OsC2DPs; the total list is shown in Supplementary Table S10. First, the results of the transcript profile revealed that during treatments of abscisic acid, gibberellic acid 3, indole-3-acetic acid, trans-zeatin, salicylic acid, and jasmonic acid, the family members exhibited diverse responses to the exogenous plant hormone-type compounds (Supplementary Figure S10). For example, *OsC2DP6* and *OsC2DP9*, among others, were consistently repressed by abscisic acid, gibberellic acid 3, indole-3-acetic acid, and trans-zeatin; *OsC2DP15* and *OsC2DP73*, among others, were consistently induced by diverse treatments; and *OsC2DP16* and *OsC2DP15*, among others, had opposing expression changes during the treatments of salicylic acid and jasmonic acid. In addition, among the three co-tandem duplications, *OsC2DP50/51/52* showed a consistent response to salicylic acid and jasmonic acid, and only *OsC2DP50/51* remained consistent during treatments of abscisic acid, gibberellic acid 3, indole-3-acetic acid, and trans-zeatin. These results suggested that OsC2DPs can be differentiated by their diverse expression patterns that possibly involved in the response to phytohormone stress; among these, the duplications might maintain similar functions or involve the same pathway for different stresses.

**Figure 5:**
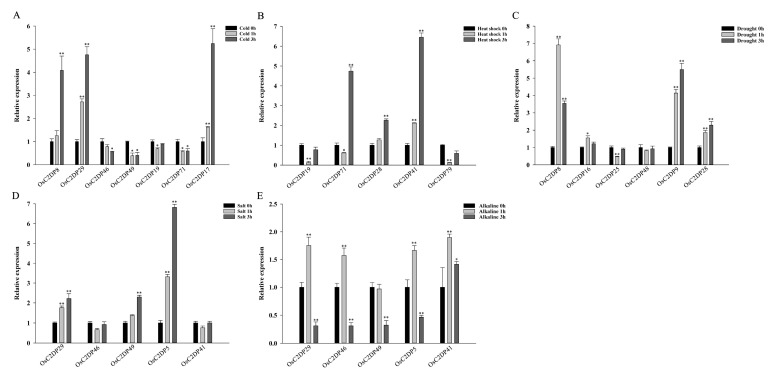
Time-course expression analysis of OsC2DPs after exposure to abiotic stress. (**A**) Cold. (**B**) Heat shock. (**C**) Drought. (**D**) Salt. (**E**) Alkaline. Data represent the mean ± SE from three replicates, asterisks indicate significant differences (Student’s *t*-test, * *p* < 0.05, ** *p* < 0.01).

Subsequently, specific abiotic-response genes were selected via previous *cis*-acting element analysis according to their total number of abiotic-related motifs. We performed qRT-PCR analysis to verify the expression changes of the candidate abiotic-response genes; the results are shown in Figure 5 and Supplementary Figure S11. The *OsC2DP8/29/17* expression was significantly induced by cold stress, the *OsC2DP46/49/71* expression was repressed, and only *OsC2DP19* showed no obvious change (Figure 5A). Under heat-shock stress, the *OsC2DP71/28/41* expression was induced, and the expression of *OsC2DP19/79* was repressed initially but subsequently recovered (Figure 5B). Such findings suggested that the *OsC2DP71* expression was induced by cold and repressed by heat, ultimately indicating this gene as a temperature-sensitive element. In addition, the *OsC2DP8/9/28* expression was induced, and the expression of *OsC2DP25* was repressed by drought stress (Figure 5C). Under salt and alkaline stress, the *OsC2DP29/5* expression was similarly induced, and the expression of *OsC2DP46/49/41* showed diverse variations in response to these two stresses (Figure 5D,E). In plants, light response is a key signal for plant growth, and both photosynthesis and circadian rhythms affect plant growth and development [32]. Therefore, we performed qRT-PCR analyses under light and dark conditions. All candidate gene expression levels showed significant variations during different treatments (Supplementary Figure S11). The *OsC2DP29/46* expression was induced, and the expression of *OsC2DP5* was repressed under dark conditions, as compared to light conditions (Supplementary Figure S11A–C). For *OsC2DP79/3/64*, circadian rhythms were observed under normal (light) growth conditions (Supplementary Figure S11D–F). In contrast, under dark conditions, the circadian rhythms of *OsC2DP79/3* were disrupted, resulting in a tapered expression (Supplementary Figure S11D,E). The *OsC2DP64* expression showed an opposite trend, as compared to that found under the normal (light) condition (Supplementary Figure S11F). These results suggested that these OsC2DPs are significantly involved in diverse responses to abiotic stress. Since *OsC2DP5/29/49/71* responded to multiple stress treatments, it could play positive or negative roles in these functions.

### 2.6. Prediction of miRNA-Targets and Enrichment Analysis of OsC2DPs

**Figure 6:**
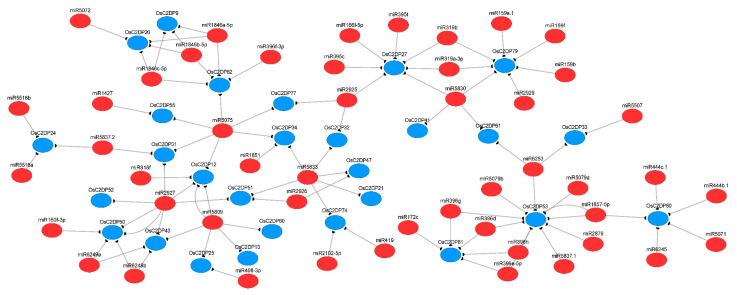
Putative regulation network for miRNA-targets of OsC2DPs. Red color and blue color circles represent miRNAs and OsC2DPs, the predicted targets are connected by solid lines.

In plants, the functions of miRNAs have been reported in diverse species, it influences plant growth, development, and support survivability under stresses environment [33], which directly acting on target gene via cleavage or translation model [34]. In this study, we analyzed the OsmiRNAs–*OsC2DPs* pathway and predicted the putative regulatory network using the miRNA database. A total of 167 unique potential OsmiRNA’s targets of OsC2DPs were identified with miRNAs 19–24 nucleotides long (Supplementary Table S11), among these, functions of 146 unique targets as the cleavage and 21 unique targets as the translation were identified, suggesting that cleavage is the major function in the regulation of OsmiRNA targets of OsC2DPs. There were 28 *OsC2DPs* involved in one relevant network (Figure 6) while the remaining 29 *OsC2DPs* were involved in fragmented networks (Supplementary Figure S12). Among these, *OsC2DP71* possessed 11 potential targeted OsmiRNAs; genes *OsC2DP53*, *OsC2DP79*, *OsC2DP27*, *OsC2DP39*, and *OsC2DP3* possessed 9, 7, 7, 7, and 6 potential targeted OsmiRNAs, respectively; and OsmiR2927, OsmiR5809, OsmiR5075, and OsmiR5833 contained six, five, six, and six potential targets, as the most and major OsmiRNA targets in the OsC2DP gene family. In addition, subfamily specific targets were identified: Group-I members, *OsC2DP27*, *OsC2DP79*, and *OsC2DP61* co-targeted by OsmiR5830; and Group-III members, *OsC2DP32*, *OsC2DP34*, *OsC2DP47*, and *OsC2DP21* co-targeted by OsmiR5833. As expected, *OsC2DP50/51/52* was co-targeted by OsmiR2927 as the co-tandem duplications, jointly targeted with Group-I members, *OsC2DP31/43/12* (Figure 6). Additionally, we performed enriched Gene Ontology (GO) and KEGG analyses of the OsC2DP gene family. Results showed major functions involved membrane, plasma membrane, multiple metabolic processes, phospholipase activity, and ion-binding of GO functions (Supplementary Figure S13A). KEGG results showed that the major pathways were membrane and multiple metabolic processes (Supplementary Figure S13B). Together, these results implied that OsC2DPs performed major functions in the cell membrane, possibly via ionic combination and transportation.

### 2.7. SNP Genotyping and Haplotype Analysis of OsC2DPs

In rice, the major subspecies japonica and indica contain different traits caused by many genotype variations; thus, we analyzed the single nucleotide polymorphism (SNP) genotyping and abiotic-related haplotypes of OsC2DPs. A total of 2861 SNPs that only exist in Japonica and Indica were selected based on diverse gene positions. The SNP annotation results showed that 424 SNPs were located in promoters; 219 SNPs were located in UTR regions; and 223 SNPs were located in exon regions, 124 of which were synonymous and 99 were non-synonymous variants (Table 2). The principal component analysis (PCA) was carried out using the OsC2DPs SNP data (only that found in Japonica and Indica varieties). The results showed that PC1 explained 87.55% of the variation and PC2 explained 12.55%, and two subspecies varieties were separated into two groups (Supplementary Figure S13C).

**Figure 7:**
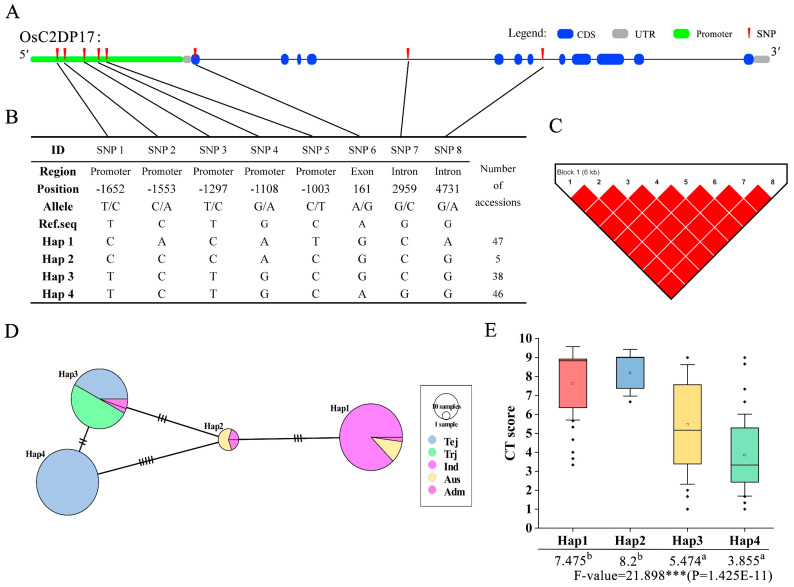
Haplotype analysis of OsC2DP17. (**A**) Structural representation of OsC2DP17 and the upstream promoter region. (**B**) OsC2DP17 SNPs and haplotype groups in 137 rice accessions. SNP positions are given relative to the start of the 5′ UTR. Hap: Haplotype. (**C**) LD analysis of OsC2DP17 using SNPs of **a**, numbers on the square represent level of LD, pure red squares represent complete LD level between each SNP. (**D**) Haplotype network variation of OsC2DP17. Circle size represents the number of accessions in each Hap, and the number of transverse lines between each Hap represents the number of nucleotide variations. Tej: Temperate Japonica; Trj: Tropical Japonica; Ind: Indica; and Adm: Admixture rice varieties. (**E**) Association of the CT score with haplotype. Different letters indicate significant differences among haplotypes (ANOVA, Duncan test), asterisks indicate significant differences in phenotype between genotypes (Student’s *t*-test, *** *p* < 0.001).

In the haplotype analysis, a set of cold-related phenotypes was associated with genotype data; this analysis was conducted to identify the functional phenotype-related genotypes in different varieties. According to previous qRT-PCR results, OsC2DP17, OsC2DP29, and OsC2DP49 were selected as candidate genes that functions related with cold stress. For OsC2DP17, after filtering genotype data for missing SNPs or heterozygotes, eight SNPs were found in the intron, exon, and promoter regions (Figure 7A). These SNPs formed four haplotypes for OsC2DP17, and the major varieties involved Haplotype1 (Hap)1 and Hap4 (Figure 7B); LD analysis results showed a strong Linkage disequilibrium (LD) relationship between each SNP pair (Figure 7C). The haplotype network and variation analysis showed that there were two major groups, Hap1 and Hap2, which contained major indica and Aus subspecies; Hap3 and Hap4 contained major temperate japonica and tropical japonica subspecies. Large genotype variations were also observed between these two groups (Figure 7D). The association of phenotype–haplotype was analyzed, and a set of cold tolerance (CT) scoring (1–9 score) was used as the evaluation index. As shown in Figure 7E, Hap1 and Hap2 showed significantly cold sensitivity (higher CT score), as compared to Hap3 and Hap4, respectively, indicating that Hap1 and Hap2 conferred major susceptibility for cold tolerance in OsC2DP17 diverse genotypes.

For OsC2DP29 and OsC2DP49, six SNPs were selected in both genes by removing missing or heterozygote data, which were found in the intron and exon regions of OsC2DP29 and the promoter, intron, and exon regions of OsC2DP49, respectively (Supplementary Figure S14A,B; Supplementary Figure S15A,B). The LD analysis of both genes revealed similar results to OsC2DP17, which had a strong LD relationship between each SNP pair (Supplementary Figures S14C and S15C). The haplotype network showed that the haplotypes of OsC2DP29 were divided into two groups: Hap1, Hap4, and Hap5, which contained the most indica, Aus, and admixture varieties (Supplementary Figure S14D); and Hap2 and Hap3, which included most tropical japonica and temperate japonica varieties. Such findings suggested that OsC2DP29 is a japonica–indica-specific genotyping gene. As expected, the phenotype-associated results showed that Hap3 and Hap2 had significantly lower CT scores than other Haps (Supplementary Figure S14E), suggesting that the genotypes of tropical japonica and temperate japonica conferred stronger cold tolerance in this population, which also aligned with the general difference in temperature sensitivity between the indica and japonica subspecies. Similarly, five haplotypes of OsC2DP49 were roughly divided into japonica (Hap1, Hap2, and Hap3) and indica (Hap4 and Hap5) groups (Supplementary Figure S15D), and only Hap3 and Hap4 contained mixture varieties. The phenotype–haplotype-associated results showed that Hap1 and Hap2 had significantly lower CT scores in populations (Supplementary Figure S15E).

These results suggested that diverse haplotypes of OsC2DP17, OsC2DP29, and OsC2DP49 could be related to cold tolerance in rice, which would require further functional demonstrations and may provide a potential theoretical foundation for the relationship between genotype variations and stress tolerance for rice populations.

## 3. Discussion

### 3.1. Identification and Phylogenetic Relationship of OsC2DPs in Rice Genome

Most C2DPs can perform functions, cooperate with other transmembrane domains such as phospholipase domain and synaptotagmin [35], and maintain highly conserved characteristics in the evolutionary process. In the present study, we used a conserved HMM model, performed a search in the rice protein database, and identified 82 OsC2DPs at each chromosome (Supplementary Table S1, Supplementary Figure S1). A few gene clusters were also found in chromosomes 1, 6, and 7. A previous study showed that gene clusters produced tandem duplication events in the genome, implying that OsC2DPs might have duplication events in cluster regions. The phylogenetic analysis revealed that a total of seven groups were divided into 82 family members according to their domains and gene structures, and multiple transmembrane-region domains were found in the different groups (Figure 2). A previous study also reported similar phylogenetic results for C2DPs; in *Arabidopsis thaliana*, the multiple C2 domains and transmembrane-region proteins were reported [27], the members of rice homologs in a previous study were similar to members of the group-III Class C in the present study; and in cotton, the rice homologs of the MCTPs were found in a phylogenetic study that were consistent with the present study [28]. In rice, a previous study identified OsNTMC2 members [29]. OsNTMC2s were shown to contain a highly conserved N-terminal TM domain, SMP domain, and C-terminal C2 domain, which was consistent with Group-VI Class A in the present study, validating the conservatism of each group and the accuracy of our analysis (Figure 2).

### 3.2. Duplications Events Performed in OsC2DPs

Normally, the duplications contain conserved domains or sequences that may involve similar functions in plants [36]. Among these, tandem duplications as a major and commonly evaluated mechanism for gene family expansion that produce novel genes and clusters into gene families had an impact on a small number of genes but had a significant contribution to gene family expansion [37]. In the present study, a total of 8, 53, 83, 74, 26, and 176 orthologous gene pairs were identified between rice and *Arabidopsis thaliana*, barley, maize, sorghum, soybean, and wheat, respectively (Supplementary Figure S6, Tables S3–S8), implying the occurrence of duplication events and the potential genome expansion of OsC2DPs from other species, especially the wheat genome. In rice, a total of six tandem and eight segmental duplications were found (Figure 3, Table 1), suggesting that these duplications might involve similar functions in rice; this hypothesis was also validated through further analysis. In the expression file data, the tandem duplications, *OsC2DP50/51/52* and *OsC2DP58/59*, showed a similar expression variation tendency with rice growth stages. Evidently, co-duplication of *OsC2DP50/51/52* expression showed the same response to diverse phytohormones and involved the same potential regulation pathway as the miRNA-targets of miR2927 (Figure 6), providing exact evidence for the predicted functions of co-duplications of *OsC2DP50/51/52*. A previous study reported that tandem duplication events occurred more frequently than other events and formed large gene copies and allelic variations [38]. Tandem duplications are widely involved in the control of stress tolerance and membrane functions in rice and *Arabidopsis thaliana* [39,40], or the transduction of the signaling pathways in legumes [41].

### 3.3. Elements Prediction and Expression Analysis of OsC2DPs

Based on the prediction of *cis*-acting elements in the promoter regions of *OsC2PDs*, many phytohormone- and abiotic stress-related motifs were found (Supplementary Figure S9), implying that OsC2DPs may be involved in phytohormones and abiotic stress. Furthermore, the expression profile data of phytohormone-type compound treatments validated the previous hypothesis, and qRT-PCR was performed to identify expression variations under abiotic stress treatments in some putative functional genes, which suggested that OsC2DPs may be involved in phytohormones response and abiotic stress. In a previous study, some OsC2DPs functions reported in transgenic plants such as OsPBP1 (LOC_Os04g44870), named OsC2DP36, involved Ca^2+^ concentration-dependent phospholipid-binding activity and were localized in the cytoplasm and the nucleus, shuttling at the plasma membrane by increased intracellular Ca^2+^ [42]. The rice no-pollen gene (LOC_Os06g40570) was named OsC2DP53 and is responsible for the production and development of pollen, which might be related to Ca^2+^ and phosphoinositol signaling pathways, depending on the C2 and GRAM domains [43]. OsC2DP (LOC_Os09g39770) was named OsC2DP78, a functional mutant that changed tolerance to salt, and showed variations in Na^+^ concentration and K^+^/Na^+^ ratio [26]. OsSMCP1 (LOC_Os07g01770) was named OsC2DP58 in present study, and each phenotype of abiotic stress was reported in a previous study by the overexpression of the transgenic line or as not mutant [25]. In addition, as mentioned above, OsC2DP58 and OsC2DP29 were tandem duplication gene pairs and contained similar expression profiles in the rice growth stage. Combined with these results, the previously reported diverse abiotic-stress-tolerance function could be considered for the study of another tandem duplication in OsC2DP59. If OsC2DP58 and OsC2DP59 involved a redundant effect, the double mutant would be a better subject on which to perform research. Due to the number of related *cis*-acting elements that were not significant in overall results, the qRT-PCR analysis did not include these target genes; this could be considered for further analysis. Taken together, except for the members of the OsNTMC2s and the MCTPs included in the previous studies, other identified OsC2DP family members that were involved in the present study also performed diverse functions in the growth development of rice.

### 3.4. Identification of Potential Variation Alleles for Future Breeding

The SNP genotyping was performed by PCA analysis using the SNP data of the OsC2DPs (Supplementary Figure S13C). The significant japonica–indica polarization of OsC2DPs supported a potential future research direction. Furthermore, diverse genotypes of the allelic variation could produce different tolerances to abiotic stress in multiple subspecies [44,45]. Haplotype analysis revealed that OsC2DP17, OsC2DP29, and OsC2DP49 possessed diverse alleles among the core collection population, which generated different cold tolerances for each variety (Figure 7, Supplementary Figures S14 and S15). We speculated that these SNPs might possess high LD levels that were coordinated with the crucial translation or functional domain region of each gene, and thereby producing a diverse phenotype in rice. In summary, these haplotypes may be potential opportunities for enhancing related tolerances in future molecular-design breeding.

## 4. Materials and Methods

### 4.1. Identification, Chromosome Distribution, and Localization of OsC2DPs

In the present study, rice genomes and protein sequences were downloaded from the Phytozome database (https://phytozome-next.jgi.doe.gov/, accessed on 15 November 2021), and the MSU Rice Genome Annotation Project database was used. To identify the OsC2DP gene family members in rice, a HMM of the C2 domain (PF00168) was downloaded from the PFAM database, version 34.0 (http://pfam.xfam.org/, accessed on 15 November 2021), which performed against the rice proteome database. With an E-value threshold less than 1e-05 [46], the filtered hits were submitted to the InterPro (https://www.ebi.ac.uk/interpro/, accessed on 15 November 2021) and SMART (http://smart.embl-heidelberg.de/, accessed on 15 November 2021) databases to search for the C2 domain again. The diverse lengths of OsC2DPs were calculated using Tbtools, version v1.098654 [47], and the physical properties (pI and *M*_W_) were analyzed using the ExPASy website (https://web.expasy.org/compute_pi/, accessed on 15 November 2021). According to the prepared gene positions obtained from the rice genome file, chromosome distribution and visualization were performed using the MapGene2Chromosome V2 (http://mg2c.iask.in/mg2c_v2.0/, accessed on 15 November 2021), according to the official default procedure.

### 4.2. Phylogenetic, Structure and Multiple Sequence Alignment Analyses

To determine the phylogenetic relationship of OsC2DPs, a phylogenetic tree was constructed using MEGA software, version 10.2 [48], and the NJ-tree function was performed by substitution model of p-distance and 1000-times bootstrap iterations were adopted. Final plot visualization was generated using tools on the iTOLs website (https://itol.embl.de/, accessed on 15 November 2021) [49]. To identify multiple structure of OsC2DPs, full-length protein sequences were submitted and analyzed using the PFAM database. Visualization was performed using each domain length and position in the website Gene Structure Display Server 2.0 (http://gsds.gao-lab.org/, accessed on 15 November 2021) [50]. Gene structures were analyzed using the software TBtools and the rice gff3 file. For multiple sequence alignment, whole protein sequences of OsC2DPs were aligned using ClustalW 2.0 [51] software and visualized using the website, Sequence Manipulation Suite (https://www.bioinformatics.org/sms2/about.html, accessed on 15 November 2021) [52], where a consistent color represents a highly conserved amino acid.

### 4.3. Synteny and Ka/Ks Analysis

To analyze synteny in OsC2DPs, C2DPs in the other six plant species were identified, the six genomes TAIR10 of *Arabidopsis thaliana*, IBSC_v2 of *Hordeum vulgare* L., Zm-B73-REFERENCE-NAM-5.0 of *Zea mays* L., Sorghum_bicolor_NCBIv3 of *Sorghum bicolor* L., Glycine_max_v2.1 of *Glycine max*, and IWGSC of *Triticum aestivum* L. were downloaded from the Ensembl genome database (https://ensembl.gramene.org/, accessed on 15 November 2021). Blastp functions were performed between the genomes of rice and those of other species using the BLAST software [53]. Thereafter, multiple genome synteny analyses were performed using MCScanX software [54]. Among these, duplications were determined by an alignment ratio over 70% (alignment length should override more than 70% of the gene length) and blast ratio over 70% (the identity region should override more than 70% of the total blast region) while tandem duplications were determined based on the involvement within a 100 kb region [55]. All duplications of C2DPs were filtered from whole genome duplications, and plots were visualized using Circos software using default parameters [56]. For non-synonymous and synonymous substitution (Ka/Ks) calculations, full-length protein sequences of all duplicated C2DPs were aligned using ClustalW 2.0. Thereafter, Ka, Ks, and Ka/Ks ratios were calculated using the software KaKs_Calculator 2.0 following the default procedure [57].

### 4.4. Expression Profile Analysis and Prediction of cis-Acting Elements

For collected expression profile data, available microarray datasets were selected from the NCBI gene expression omnibus (GEO) database. The rice-tissue-specific expression and duplication expression pattern data followed accession number GSE19024 [58], exogenous phytohormones-type treatments of abscisic acid (3 h/50 µM), gibberellic acid 3 (3 h/10 µM), indole-3-acetic acid (3 h/10 µM), trans-zeatin (3 h/1 µM), salicylic acid (3 h/100 µM), and jasmonic acid (3 h/100 µM) data followed accession numbers GSE39429 and GSE37557 [59,60]. After the OsC2DP expression data were filtered from these data profiles, the mean value of replications and related fold change were calculated by comparison to the control (no treatment). Visualization of the heatmap was performed using the R program (Version: 3.6.3), and data were analyzed using log10-transformed, row normalization, and cluster processing. For prediction of *cis*-acting elements for OsC2DPs, all promoters of OsC2DPs were extracted using the software, TBtools, and the promoter was determined using 2000 bp upstream sequences of the transcription initiation site for each family member. Element prediction was performed using the database, PlantCARE [61], and the related motifs were classified according to database annotations.

### 4.5. Plant Growth, Treatment, and Quantitative Real-Time PCR Analysis

To validate the OsC2DP expression response to abiotic stress, common variety Nipponbare (IT003166) was selected for the experiment, which was obtained from Rural Development Administration (RDA) National Institute of Agricultural Sciences (http://genebank.rda.go.kr/initMain.do, accessed on 15 November 2021). Plants were grown to 2-leaf stage seedlings using Yoshida growth solutions under normal growth conditions (28/26 °C, 12/12 h, 65% relative humidity, light/300 μmol m^−2^ s^−1^). Thereafter, they were collected under treatments of cold (4 °C), heat (42 °C), drought (dehydration), salt (200 mM), and alkaline (0.15% Na_2_CO_3_) stress. For light response, Nipponbare plants were grown to the 2-leaf stage and leaf samples were collected under the different conditions. The control group followed normal growth conditions, and treatment group was the same as the control but treated always in dark conditions, and the collected leaf sample followed time points. The time points for collection were 0, 1, and 3 h for cold, heat shock, drought, salt, and alkaline stress; and 0, 12, 24, 36, and 48 h for light and dark treatments. Total RNA was extracted from the collected leaf samples using the RNeasy Plant Mini Kit (QIAGEN). All samples were treated with RNase-Free DNase (QIAGEN) to remove genomic DNA. Complementary DNA was synthesized using the SuperScript III Kit (Thermo). For the primer design, each gene cds was inputted and produced by the software, Primer5. The gene, *Actin*, served as the internal control to ensure corrected accuracy of results. A qRT-PCR was performed using SYBR Green PCR Kit (QIAGEN) and Rotor-Gene Q (QIAGEN) system, following the default production procedure. Each reaction was repeated three times; the mean value was calculated and the 2^−ΔΔCt^ method was used to calculate the relative expression [62].

### 4.6. miRNA Target Prediction and Enrichment Analysis of OsC2DPs

To predict the potential miRNA targets of OsC2DPs, the full lengths of the cds of OsC2DPs were extracted and predicted using the miRNA database, psRNATarget [63]. The expectation threshold was set to 4 for filtered pseudo results, and visualization was performed using the Cytoscape software [64]. For GO and KEGG enrichment, the PlantGSEA database (http://bioinformatics.cau.edu.cn/PlantGSEA/, accessed on 15 November 2021) was employed for OsC2DPs [65]. The results were filtered by adjusted *p*-values less than 0.05, showing 14 major enrichment functional terms, and visualization was performed using the R program.

### 4.7. SNP Genotyping and Haplotype Analysis

A core collection set of rice was employed in the present study. A total of 137 varieties containing multiple major rice subspecies including temperate japonica (Tej); tropical japonica (Trj); indica (Ind); and aus and admixture (Adm) were collected from 28 countries [66]. Approximately 1.85 million high-quality SNPs were filtered from raw re-sequencing data, according to a previous study [67]. For SNP genotyping of OsC2DPs, only japonica and indica SNPs located at the promoter, UTR, intron, and exon of OsC2DPs formed a 2861 SNP set; the annotations were performed using website tools http://bioinfo.sibs.ac.cn/, accessed on 15 November 2021. PCA and visualizations were performed using R program default functions using 2861 SNPs data. For haplotype analysis, all SNPs located at OsC2DP17, OsC2DP29, and OsC2DP49 were collected following the above descriptions, and the missing and heterozygote data were filtered. Phenotype data included an evaluation of the CT score, according to a previous study [68]. Haplotype grouping was carried out based on the SNP variations between each Hap. Significant tests of phenotype–haplotype was carried out using SPSS software; different letters represent differences. Visualization of the haplotype network was performed using the software PopArt [69]. LD analysis was performed using Haploview software [70].

## 5. Conclusions

Herein, 82 OsC2DP gene family members and multiple transmembrane domains were identified. The roles of the OsC2DP genes in abiotic stress were discovered by bioinformatics prediction, transcript profile analysis, and qRT-PCR analysis. Furthermore, the haplotype analysis also provided evidence to identify the role of OsC2DPs in abiotic stress. In summary, we have provided the first study to comprehensively characterize the C2DP gene family in rice, and the findings of this study contribute to a broader understanding of the functions of the C2 domain and the future functional characterizations of OsC2DPs.

## Figures and Tables

**Table 1:** Synteny analysis for OsC2DP gene family intra rice and rice genome.

Tandem Duplication										Ka	Ks	Ka/Ks	S	N	Effective Len
Gene Symbol	Gene ID	Chr.	Start	End	Gene Symbol	Gene ID	Chr.	Start	End						
OsC2DP6	LOC_Os01g60340	Chr1	34,902,303	34,906,955	OsC2DP7	LOC_Os01g60350	Chr1	34,908,079	34,908,079	0.15904	0.34162	0.46554	326.167	1161.83	1488
OsC2DP50	LOC_Os06g40170	Chr6	23,908,918	23,913,797	OsC2DP51	LOC_Os06g40180	Chr6	23,921,940	23,921,940	0.09049	0.14449	0.62625	590.333	1893.67	2484
OsC2DP51	LOC_Os06g40180	Chr6	23,921,940	23,924,921	OsC2DP52	LOC_Os06g40190	Chr6	23,928,702	23,928,702	0.16077	0.48728	0.32993	590.667	1854.33	2445
OsC2DP50	LOC_Os06g40170	Chr6	23,908,918	23,913,797	OsC2DP52	LOC_Os06g40190	Chr6	23,928,702	23,928,702	0.16616	0.45379	0.36617	588.167	1856.83	2445
OsC2DP58	LOC_Os07g01770	Chr7	452,093	460,151	OsC2DP59	LOC_Os07g01780	Chr7	457,738	457,738	0.00405	0.00448	0.90514	111.975	371.025	483
OsC2DP67	LOC_Os07g47390	Chr7	28,339,687	28,340,470	OsC2DP68	LOC_Os07g47400	Chr7	28,344,133	28,344,133	0.40704	0.60314	0.67487	190.833	493.167	684
**Segmental Duplication**										**Ka**	**Ks**	**Ka/Ks**	**S**	**N**	**Effective Len**
**Gene Symbol**	**Gene ID**	**Chr.**	**Start**	**End**	**Gene Symbol**	**Gene ID**	**Chr.**	**Start**	**End**						
OsC2DP11	LOC_Os01g72230	Chr1	41,882,488	41,886,410	OsC2DP46	LOC_Os05g31720	Chr5	18,466,963	18,470,649	0.26074	1.11476	0.2339	246.833	875.167	1122
OsC2DP2	LOC_Os01g07760	Chr1	3,724,314	3,729,284	OsC2DP43	LOC_Os05g07880	Chr5	4,255,763	4,260,333	0.11203	5.56155	0.02014	396.932	2039.07	2436
OsC2DP10	LOC_Os01g70790	Chr1	40,973,255	40,974,782	OsC2DP67	LOC_Os07g47390	Chr7	28,339,687	28,340,470	0.40741	0.63562	0.64096	190.917	484.083	675
OsC2DP20	LOC_Os02g42710	Chr2	25,684,044	25,687,149	OsC2DP36	LOC_Os04g44870	Chr4	26,558,810	26,561,326	0.1123	0.57604	0.19495	97	326	423
OsC2DP13	LOC_Os02g10480	Chr2	5,505,016	5,508,425	OsC2DP54	LOC_Os06g40704	Chr6	24,264,404	24,268,015	0.15437	0.84849	0.18193	217.5	727.5	945
OsC2DP14	LOC_Os02g10630	Chr2	5,586,939	5,593,645	OsC2DP53	LOC_Os06g40570	Chr6	24,187,249	24,192,151	0.22964	0.83077	0.27643	664.167	2266.83	2931
OsC2DP30	LOC_Os03g18010	Chr3	10,025,401	10,027,931	OsC2DP69	LOC_Os07g49330	Chr7	29,545,825	29,548,981	0.17977	1.14005	0.15769	325.667	1108.33	1434
OsC2DP72	LOC_Os08g44850	Chr8	28,170,358	28,172,330	OsC2DP78	LOC_Os09g39770	Chr9	22,805,610	22,807,049	0.16323	0.41331	0.39494	231.833	602.167	834

**Table 2:** Summary for SNPs in OsC2DPs.

Variation Type	Count	Variation in Exon	Count
Promoter variation	424		
Upstream variation	575		
Downstream variation	73		
Upstream and downstream variation	265		
5’UTR variation	47		
3’UTR variation	172		
Intergenic variation	178		
Exonic variation	223	Synonymous variant	124
		Non-synonymous variant	99
Intronic variation	829		
ncRNA_exonic variation	75		

## Data Availability

The genome, protein, and Generic Feature Format files of rice, *Arabidopsis thaliana*, *Hordeum vulgare* L., *Zea mays* L., *Sorghum bicolor* L., *Glycine max*, and *Triticum aestivum* L. were downloaded from the Phytozome database (https://phytozome-next.jgi.doe.gov/, accessed on 15 November 2021) and Ensemble FTP (http://plants.ensembl.org/info/data/ftp/index.html, accessed on 15 November 2021). The microarray data generated during the current study have been deposited and publicly available at the National Center of Biotechnology Information repository, GEO database (https://www.ncbi.nlm.nih.gov/geo/, accessed on 15 November 2021), accession numbers: GSE19024, GSE39429 and GSE37557.

## Supplementary Materials

The supporting information can be downloaded at: https://www.mdpi.com/article/10.3390/ijms23042221/s1.

## References

[1] Kirkby E., Pilbeam D. (1984). Calcium as a plant nutrient. Plant Cell Environ..

[2] Du L., Ali G.S., Simons K.A., Hou J., Yang T., Reddy A., Poovaiah B. (2009). Ca^2+^/calmodulin regulates salicylic-acid-mediated plant immunity. Nature.

[3] Kudla J., Batistič O., Hashimoto K. (2010). Calcium signals: The lead currency of plant information processing. Plant Cell.

[4] Sanders D., Brownlee C., Harper J.F. (1999). Communicating with calcium. Plant Cell.

[5] Xiong L., Schumaker K.S., Zhu J.-K. (2002). Cell signaling during cold, drought, and salt stress. Plant Cell.

[6] Luan S., Kudla J., Rodriguez-Concepcion M., Yalovsky S., Gruissem W. (2002). Calmodulins and calcineurin B–like proteins: Calcium sensors for specific signal response coupling in plants. Plant Cell.

[7] Plant P.J., Yeger H., Staub O., Howard P., Rotin D. (1997). The C2 Domain of the Ubiquitin Protein Ligase Nedd4 Mediates Ca^2+^-dependent Plasma Membrane Localization. J. Biol. Chem..

[8] Farah C.A., Sossin W.S. (2012). The role of C2 domains in PKC signaling. Calcium Signal..

[9] Kopka J., Pical C., Hetherington A.M., Müller-Röber B. (1998). Ca^2+^/phospholipid-binding (C2) domain in multiple plant proteins: Novel components of the calcium-sensing apparatus. Plant Mol. Biol..

[10] Merithew E., Lambright D.G. (2002). Calculating the potential of C2 domains for membrane binding. Dev. Cell.

[11] Schapire A.L., Voigt B., Jasik J., Rosado A., Lopez-Cobollo R., Menzel D., Salinas J., Mancuso S., Valpuesta V., Baluska F. (2008). Arabidopsis synaptotagmin 1 is required for the maintenance of plasma membrane integrity and cell viability. Plant Cell.

[12] Molz L., Chen Y.-W., Hirano M., Williams L.T. (1996). Cpk is a novel class of Drosophila PtdIns 3-kinase containing a C2 domain. J. Biol. Chem..

[13] Maeda I., Kohara Y., Yamamoto M., Sugimoto A. (2001). Large-scale analysis of gene function in Caenorhabditis elegans by high-throughput RNAi. Curr. Biol..

[14] Cho W., Stahelin R.V. (2006). Membrane binding and subcellular targeting of C2 domains. Biochim. Et Biophys. Acta (BBA) Mol. Cell Biol. Lipids.

[15] Nalefski E.A., Falke J.J. (1996). The C2 domain calcium-binding motif: Structural and functional diversity. Protein Sci..

[16] Zhai X., Gao Y.-G., Boldyrev I.A., Malinina L., Patel D.J., Molotkovsky J.G., Chalfant C.A., Brown R.E. (2017). Regulation of Membrane Binding by the C2-Domain of Cytoplasmic Phospholipase A2 by Ceramide-1-Phosphate and Calcium. Biophys. J..

[17] Zhang X., Jiang S., Mitok K.A., Li L., Attie A.D., Martin T.F. (2017). BAIAP3, a C2 domain–containing Munc13 protein, controls the fate of dense-core vesicles in neuroendocrine cells. J. Cell Biol..

[18] Souroujon M.C., Mochly-Rosen D. (1998). Peptide modulators of protein–protein interactions in intracellular signaling. Nat. Biotechnol..

[19] Brandman R., Disatnik M.-H., Churchill E., Mochly-Rosen D. (2006). Peptides derived from the C2 domain of protein kinase C epsilon (epsilon PKC) modulate epsilon PKC activity and identify potential protein-protein interaction surfaces. J. Biol. Chem..

[20] Rahier R., Noiriel A., Abousalham A. (2016). Functional characterization of the N-Terminal C2 domain from Arabidopsis thaliana phospholipase Dα and Dβ. BioMed Res. Int..

[21] Premkumar A., Lindberg S., Lager I., Rasmussen U., Schulz A. (2019). Arabidopsis PLDs with C2-domain function distinctively in hypoxia. Physiol. Plant.

[22] Cheung M.-Y., Ngo J.C.-K., Chen Z., Jia Q., Li T., Gou Y., Wang Y., Lam H.-M. (2020). A structure model explaining the binding between a ubiquitous unconventional G-protein (OsYchF1) and a plant-specific C2-domain protein (OsGAP1) from rice. Biochem. J..

[23] Vaddepalli P., Herrmann A., Fulton L., Oelschner M., Hillmer S., Stratil T.F., Fastner A., Hammes U.Z., Ott T., Robinson D.G. (2014). The C2-domain protein QUIRKY and the receptor-like kinase STRUBBELIG localize to plasmodesmata and mediate tissue morphogenesis in Arabidopsis thaliana. Development.

[24] Kim C.Y., Koo Y.D., Jin J.B., Moon B.C., Kang C.H., Kim S.T., Park B.O., Lee S.Y., Kim M.L., Hwang I. (2003). Rice C2-domain proteins are induced and translocated to the plasma membrane in response to a fungal elicitor. Biochemistry.

[25] Yokotani N., Ichikawa T., Kondou Y., Maeda S., Iwabuchi M., Mori M., Hirochika H., Matsui M., Oda K. (2009). Overexpression of a rice gene encoding a small C2 domain protein OsSMCP1 increases tolerance to abiotic and biotic stresses in transgenic Arabidopsis. Plant Mol. Biol..

[26] Fu S., Fu L., Zhang X., Huang J., Yang G., Wang Z., Liu Y.-G., Zhang G., Wu D., Xia J. (2019). OsC2DP, a novel C2 domain-containing protein is required for salt tolerance in rice. Plant Cell Physiol..

[27] Liu L., Li C., Liang Z., Yu H. (2018). Characterization of multiple C2 domain and transmembrane region proteins in Arabidopsis. Plant Physiol..

[28] Hao P., Wang H., Ma L., Wu A., Chen P., Cheng S., Wei H., Yu S. (2020). Genome-wide identification and characterization of multiple C2 domains and transmembrane region proteins in Gossypium hirsutum. BMC Genom..

[29] Huang R., Zhao J., Liu J., Wang Y., Han S., Zhao H. (2017). Genome-wide analysis and expression profiles of NTMC2 family genes in Oryza sativa. Gene.

[30] Hammoudi V., Vlachakis G., Schranz M.E., van den Burg H.A. (2016). Whole-genome duplications followed by tandem duplications drive diversification of the protein modifier SUMO in Angiosperms. New Phytol..

[31] Chow C.-N., Chiang-Hsieh Y.-F., Chien C.-H., Zheng H.-Q., Lee T.-Y., Wu N.-Y., Tseng K.-C., Hou P.-F., Chang W.-C. (2018). Delineation of condition specific Cis-and Trans-acting elements in plant promoters under various Endo-and exogenous stimuli. BMC Genom..

[32] Lu X., Song S., Xiao Y., Fan F., Zhou Y., Jia G., Tang W., Peng J. (2021). Circadian clock-coordinated response to chilling stress in rice. Environ. Exp. Bot..

[33] Zheng L., Zhang C., Shi C., Wang Y., Zhou T., Sun F., Wang H., Zhao S., Qin Q., Qiao R. (2017). Rice stripe virus NS3 protein regulates primary miRNA processing through association with the miRNA biogenesis factor OsDRB1 and facilitates virus infection in rice. PLoS Pathog..

[34] Archak S., Nagaraju J. (2007). Computational prediction of rice (*Oryza sativa*) miRNA targets. Genom. Proteom. Bioinform..

[35] Nalefski E.A., Wisner M.A., Chen J.Z., Sprang S.R., Fukuda M., Mikoshiba K., Falke J.J. (2001). C2 domains from different Ca^2+^ signaling pathways display functional and mechanistic diversity. Biochemistry.

[36] Leister D. (2004). Tandem and segmental gene duplication and recombination in the evolution of plant disease resistance genes. Trends Genet..

[37] Liu C., Wu Y., Liu Y., Yang L., Dong R., Jiang L., Liu P., Liu G., Wang Z., Luo L. (2021). Genome-wide analysis of tandem duplicated genes and their contribution to stress resistance in pigeonpea (*Cajanus cajan*). Genomics.

[38] Yu J., Ke T., Tehrim S., Sun F., Liao B., Hua W. (2015). PTGBase: An integrated database to study tandem duplicated genes in plants. Database.

[39] Clark R.M., Schweikert G., Toomajian C., Ossowski S., Zeller G., Shinn P., Warthmann N., Hu T.T., Fu G., Hinds D.A. (2007). Common sequence polymorphisms shaping genetic diversity in Arabidopsis thaliana. Science.

[40] Rizzon C., Ponger L., Gaut B.S. (2006). Striking similarities in the genomic distribution of tandemly arrayed genes in Arabidopsis and rice. PLoS Comput. Biol..

[41] Bellieny-Rabelo D., Oliveira A.E.A., Venancio T.M. (2013). Impact of whole-genome and tandem duplications in the expansion and functional diversification of the F-box family in legumes (Fabaceae). PLoS ONE.

[42] Yang W.-Q., Lai Y., Li M.-N., Xu W.-Y., Xue Y.-B. (2008). A novel C2-domain phospholipid-binding protein, OsPBP1, is required for pollen fertility in rice. Mol. Plant.

[43] Jiang S.Y., Cai M., Ramachandran S. (2005). The Oryza sativa no pollen (Osnop) gene plays a role in male gametophyte development and most likely encodes a C2-GRAM domain-containing protein. Plant Mol. Biol..

[44] Liu C., Ou S., Mao B., Tang J., Wang W., Wang H., Cao S., Schläppi M.R., Zhao B., Xiao G. (2018). Early selection of bZIP73 facilitated adaptation of japonica rice to cold climates. Nat. Commun..

[45] Liu C., Schläppi M.R., Mao B., Wang W., Wang A., Chu C. (2019). The bZIP 73 transcription factor controls rice cold tolerance at the reproductive stage. Plant Biotechnol. J..

[46] Fjell C.D., Jenssen H., Fries P., Aich P., Griebel P., Hilpert K., Hancock R.E., Cherkasov A. (2008). Identification of novel host defense peptides and the absence of α-defensins in the bovine genome. Proteins Struct. Funct. Bioinf..

[47] Chen C., Chen H., Zhang Y., Thomas H.R., Frank M.H., He Y., Xia R. (2020). TBtools: An integrative toolkit developed for interactive analyses of big biological data. Mol. Plant.

[48] Kumar S., Stecher G., Li M., Knyaz C., Tamura K. (2018). MEGA X: Molecular evolutionary genetics analysis across computing platforms. Mol. Biol. Evol..

[49] Letunic I., Bork P. (2019). Interactive Tree of Life (iTOL) v4: Recent updates and new developments. Nucleic Acids Res..

[50] Hu B., Jin J., Guo A.-Y., Zhang H., Luo J., Gao G. (2015). GSDS 2.0: An upgraded gene feature visualization server. Bioinformatics.

[51] Larkin M.A., Blackshields G., Brown N.P., Chenna R., McGettigan P.A., McWilliam H., Valentin F., Wallace I.M., Wilm A., Lopez R. (2007). Clustal W and Clustal X version 2.0. Bioinformatics.

[52] Stothard P. (2000). The sequence manipulation suite: JavaScript programs for analyzing and formatting protein and DNA sequences. Biotechniques.

[53] Altschul S.F., Gish W., Miller W., Myers E.W., Lipman D.J. (1990). Basic local alignment search tool. J. Mol. Biol..

[54] Wang Y., Tang H., DeBarry J.D., Tan X., Li J., Wang X., Lee T.-h., Jin H., Marler B., Guo H. (2012). MCScanX: A toolkit for detection and evolutionary analysis of gene synteny and collinearity. Nucleic Acids Res..

[55] Cui L., Yang G., Yan J., Pan Y., Nie X. (2019). Genome-wide identification, expression profiles and regulatory network of MAPK cascade gene family in barley. BMC Genom..

[56] Krzywinski M., Schein J., Birol I., Connors J., Gascoyne R., Horsman D., Jones S.J., Marra M.A. (2009). Circos: An information aesthetic for comparative genomics. Genome Res..

[57] Wang D., Zhang Y., Zhang Z., Zhu J., Yu J. (2010). KaKs_Calculator 2.0: A toolkit incorporating gamma-series methods and sliding window strategies. Genom. Proteom. Bioinform..

[58] Wang L., Xie W., Chen Y., Tang W., Yang J., Ye R., Liu L., Lin Y., Xu C., Xiao J. (2010). A dynamic gene expression atlas covering the entire life cycle of rice. Plant J..

[59] Garg R., Tyagi A.K., Jain M. (2012). Microarray analysis reveals overlapping and specific transcriptional responses to different plant hormones in rice. Plant Signal. Behav..

[60] Sato Y., Takehisa H., Kamatsuki K., Minami H., Namiki N., Ikawa H., Ohyanagi H., Sugimoto K., Antonio B.A., Nagamura Y. (2013). RiceXPro version 3.0: Expanding the informatics resource for rice transcriptome. Nucleic Acids Res..

[61] Lescot M., Déhais P., Thijs G., Marchal K., Moreau Y., Van de Peer Y., Rouzé P., Rombauts S. (2002). PlantCARE, a database of plant cis-acting regulatory elements and a portal to tools for in silico analysis of promoter sequences. Nucleic Acids Res..

[62] Rao X., Huang X., Zhou Z., Lin X. (2013). An improvement of the 2^−ΔΔCT^ method for quantitative real-time polymerase chain reaction data analysis. Biostat. Bioinform. Biomath..

[63] Dai X., Zhao P.X. (2011). psRNATarget: A plant small RNA target analysis server. Nucleic Acids Res..

[64] Shannon P., Markiel A., Ozier O., Baliga N.S., Wang J.T., Ramage D., Amin N., Schwikowski B., Ideker T. (2003). Cytoscape: A software environment for integrated models of biomolecular interaction networks. Genome Res..

[65] Yi X., Du Z., Su Z. (2013). PlantGSEA: A gene set enrichment analysis toolkit for plant community. Nucleic Acids Res..

[66] Kim T.-S., He Q., Kim K.-W., Yoon M.-Y., Ra W.-H., Li F.P., Tong W., Yu J., Oo W.H., Choi B. (2016). Genome-wide resequencing of KRICE_CORE reveals their potential for future breeding, as well as functional and evolutionary studies in the post-genomic era. BMC Genom..

[67] Zhang H., San M.L., Jang S.-G., Lee J.-H., Kim N.-E., Lee A.-R., Park S.-Y., Cao F.-Y., Chin J.-H., Kwon S.-W. (2020). Genome-Wide Association Study of Root System Development at Seedling Stage in Rice. Genes.

[68] Zhang H., Wu T., Li Z., Huang K., Kim N.-E., Ma Z., Kwon S.-W., Jiang W., Du X. (2021). OsGATA16, a GATA Transcription Factor, Confers Cold Tolerance by Repressing OsWRKY45–1 at the Seedling Stage in Rice. Rice.

[69] Leigh J.W., Bryant D. (2015). popart: Full-feature software for haplotype network construction. Methods Ecol. Evol..

[70] Barrett J.C., Fry B., Maller J., Daly M.J. (2005). Haploview: Analysis and visualization of LD and haplotype maps. Bioinformatics.

