# Supplementary figures and images for: Global Identification and Characterization of C2 Domain-Containing Proteins Associated with Abiotic Stress Response in Rice (Oryza sativa L.)
Source: Int J Mol Sci. 2022 Feb 17;23(4):2221. doi: 10.3390/ijms23042221 (PMC8875736; doi:10.3390/ijms23042221)

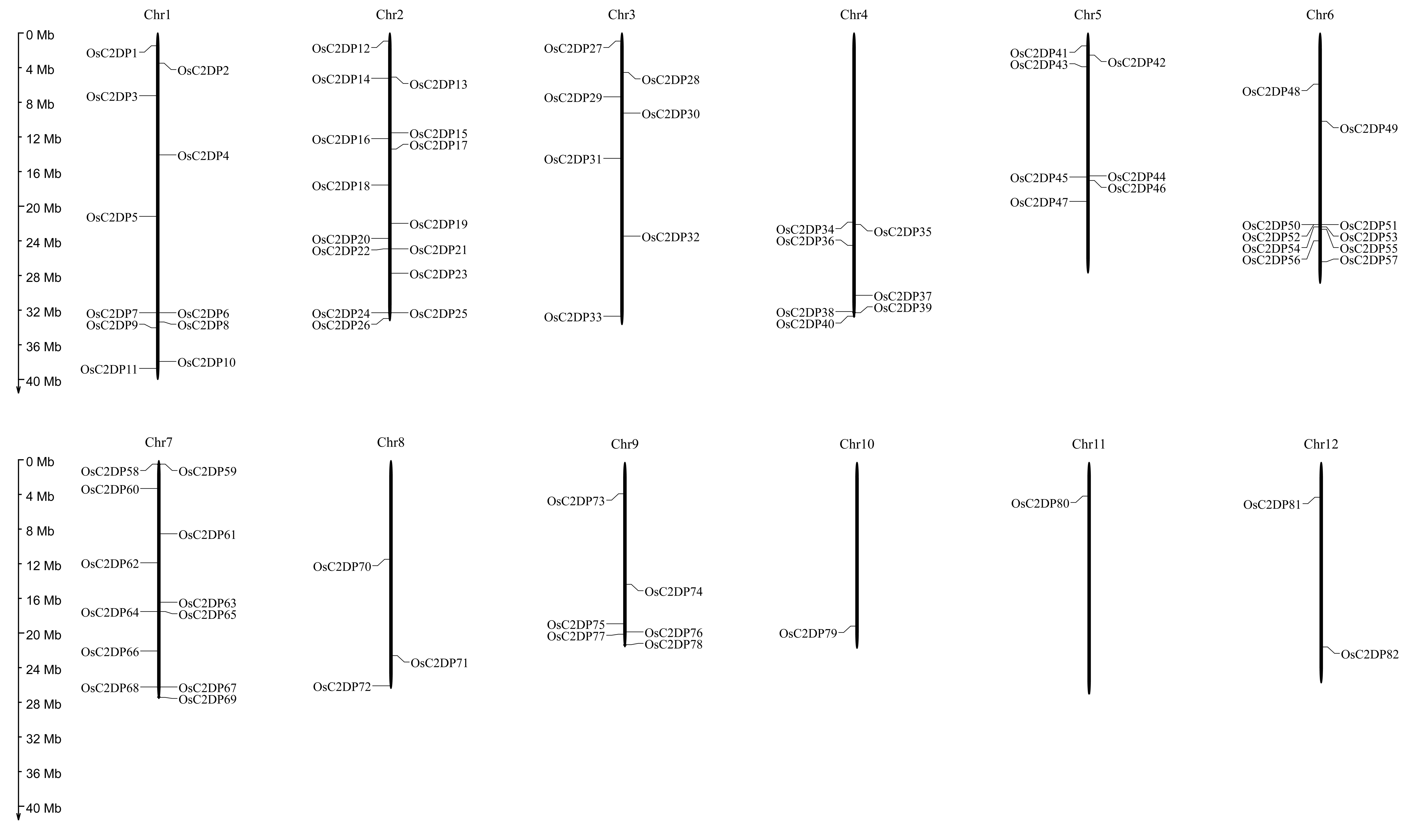

Supplement: Supplementary file 1 [file ijms-23-02221-s001.zip › ijms-1596146-supplementary/Figure S1.jpg]

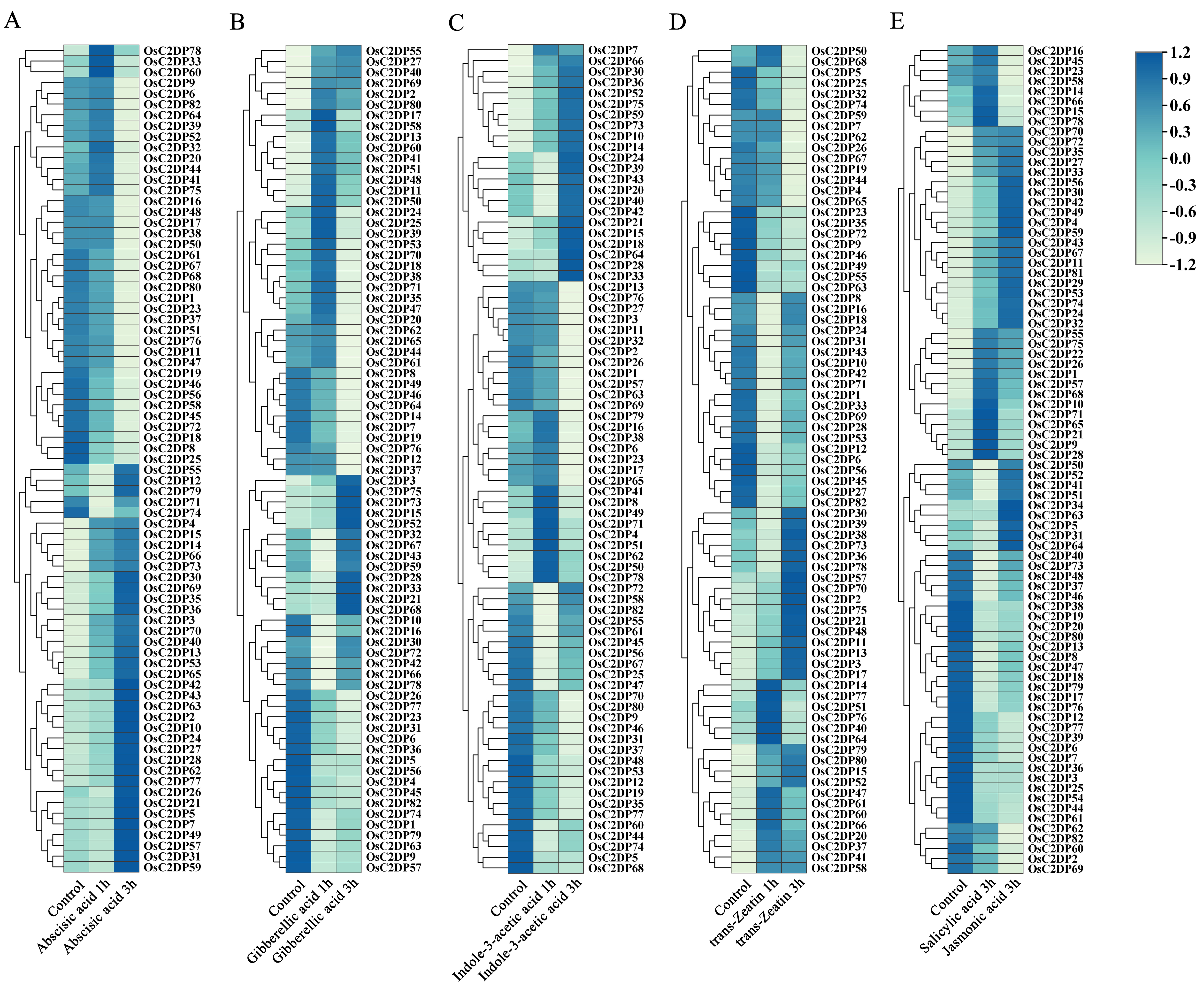

Supplement: Supplementary file 1 [file ijms-23-02221-s001.zip › ijms-1596146-supplementary/Figure S10.jpg]

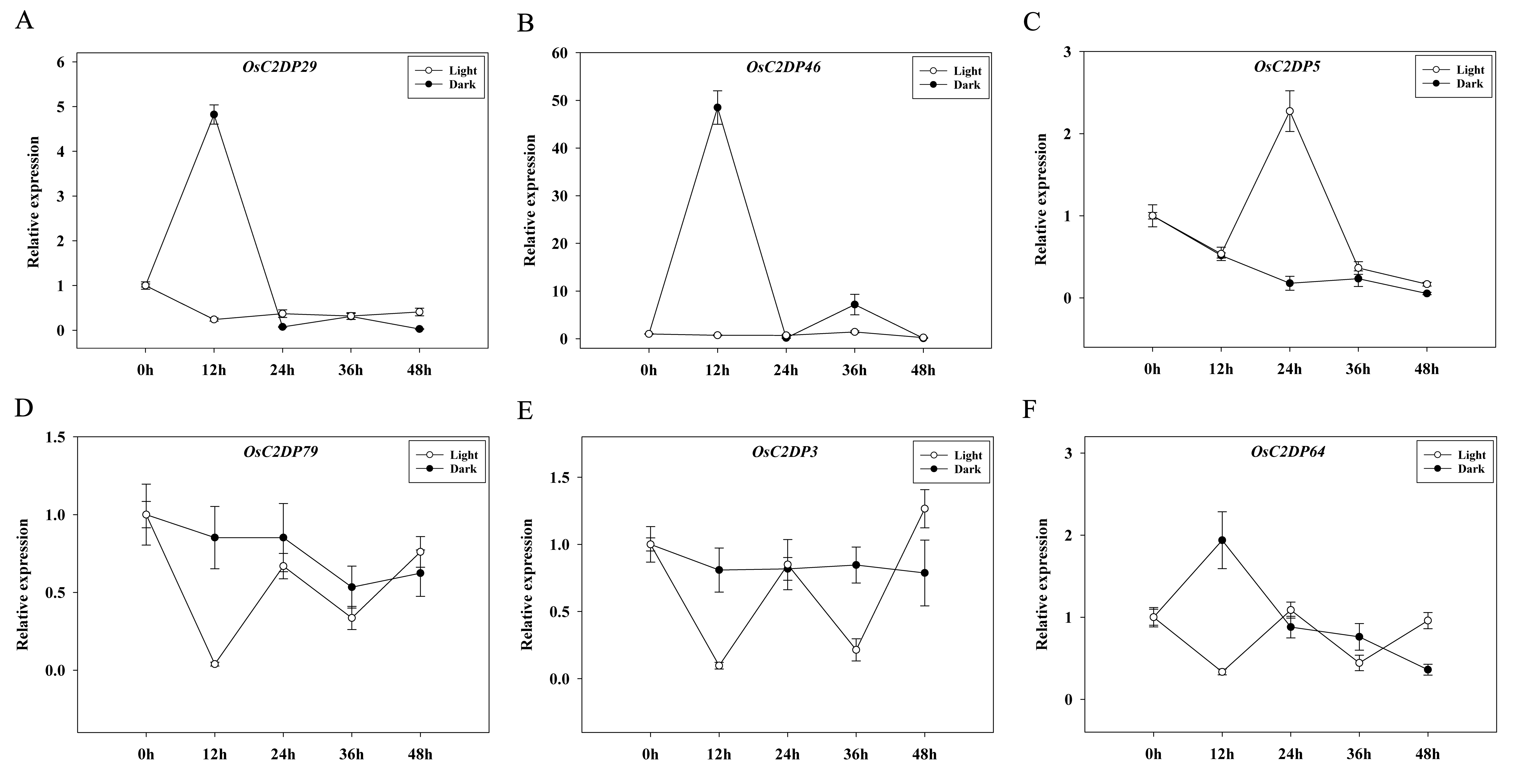

Supplement: Supplementary file 1 [file ijms-23-02221-s001.zip › ijms-1596146-supplementary/Figure S11.jpg]

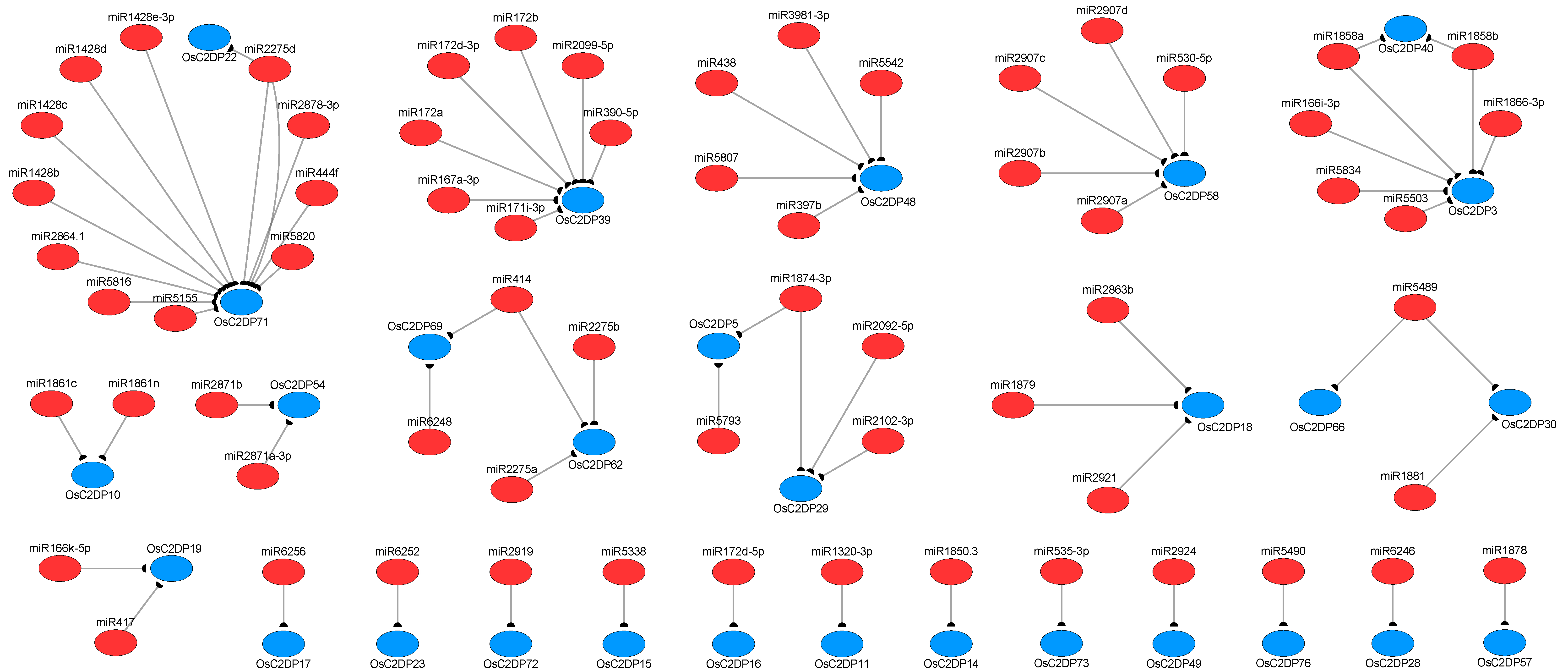

Supplement: Supplementary file 1 [file ijms-23-02221-s001.zip › ijms-1596146-supplementary/Figure S12.jpg]

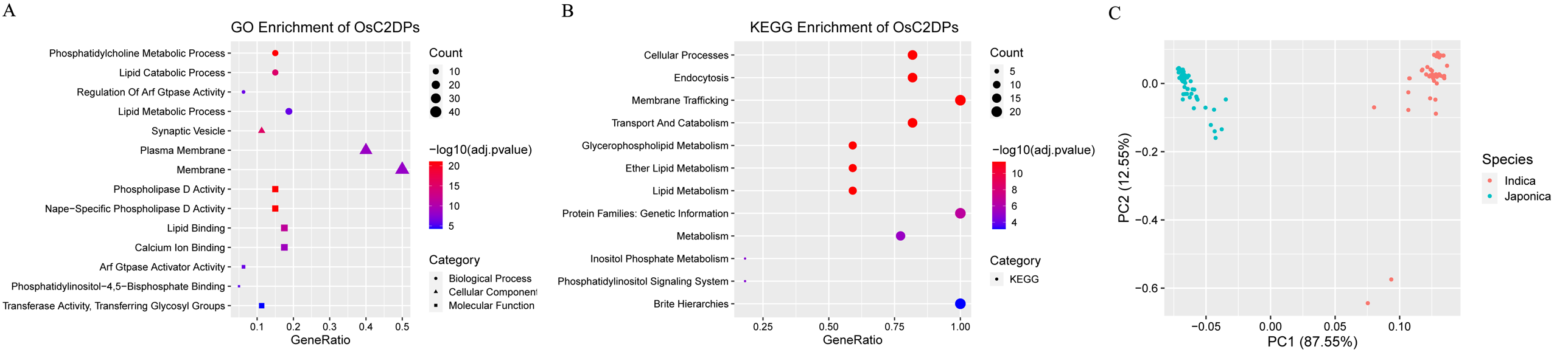

Supplement: Supplementary file 1 [file ijms-23-02221-s001.zip › ijms-1596146-supplementary/Figure S13.jpg]

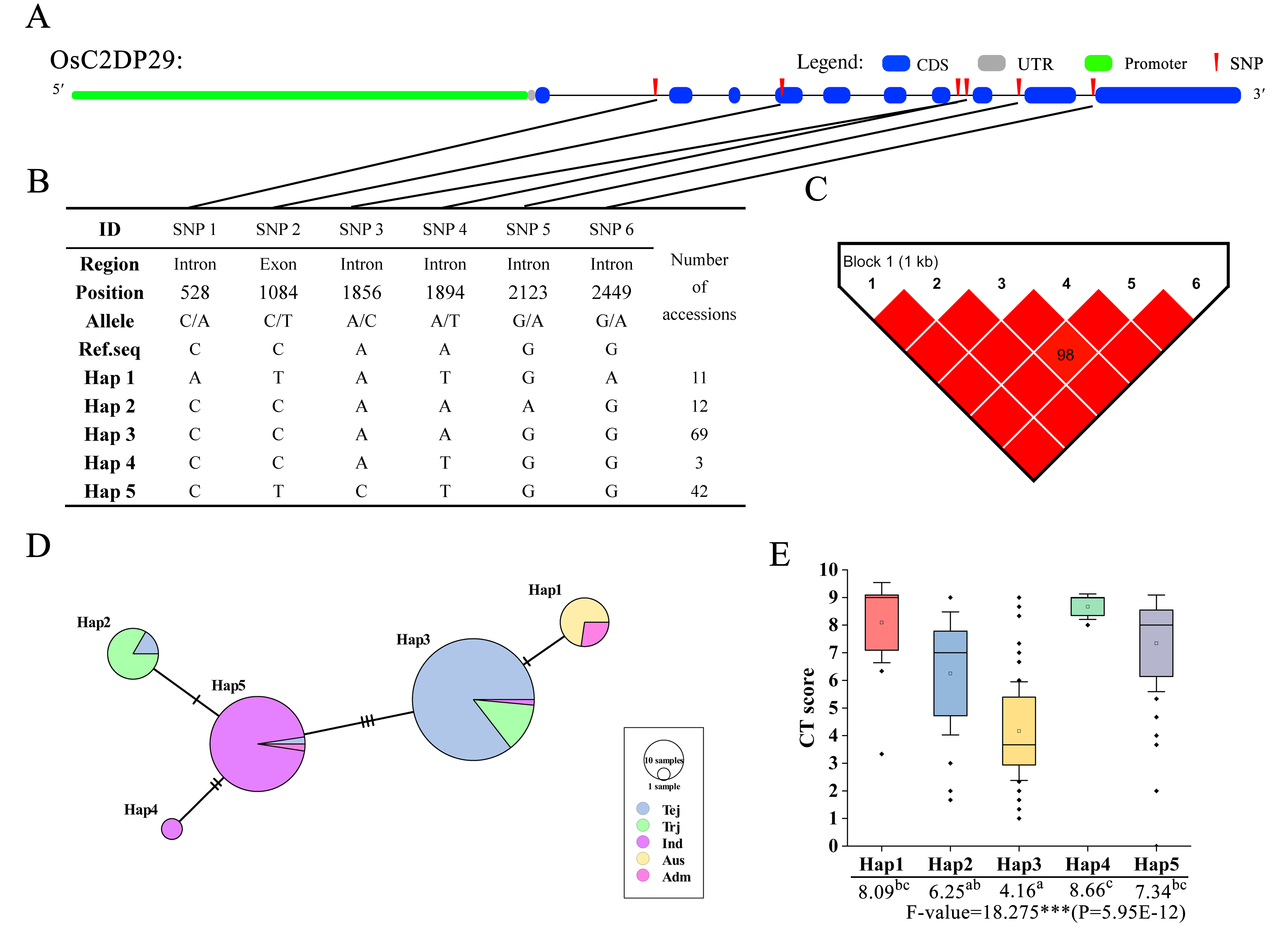

Supplement: Supplementary file 1 [file ijms-23-02221-s001.zip › ijms-1596146-supplementary/Figure S14.jpg]

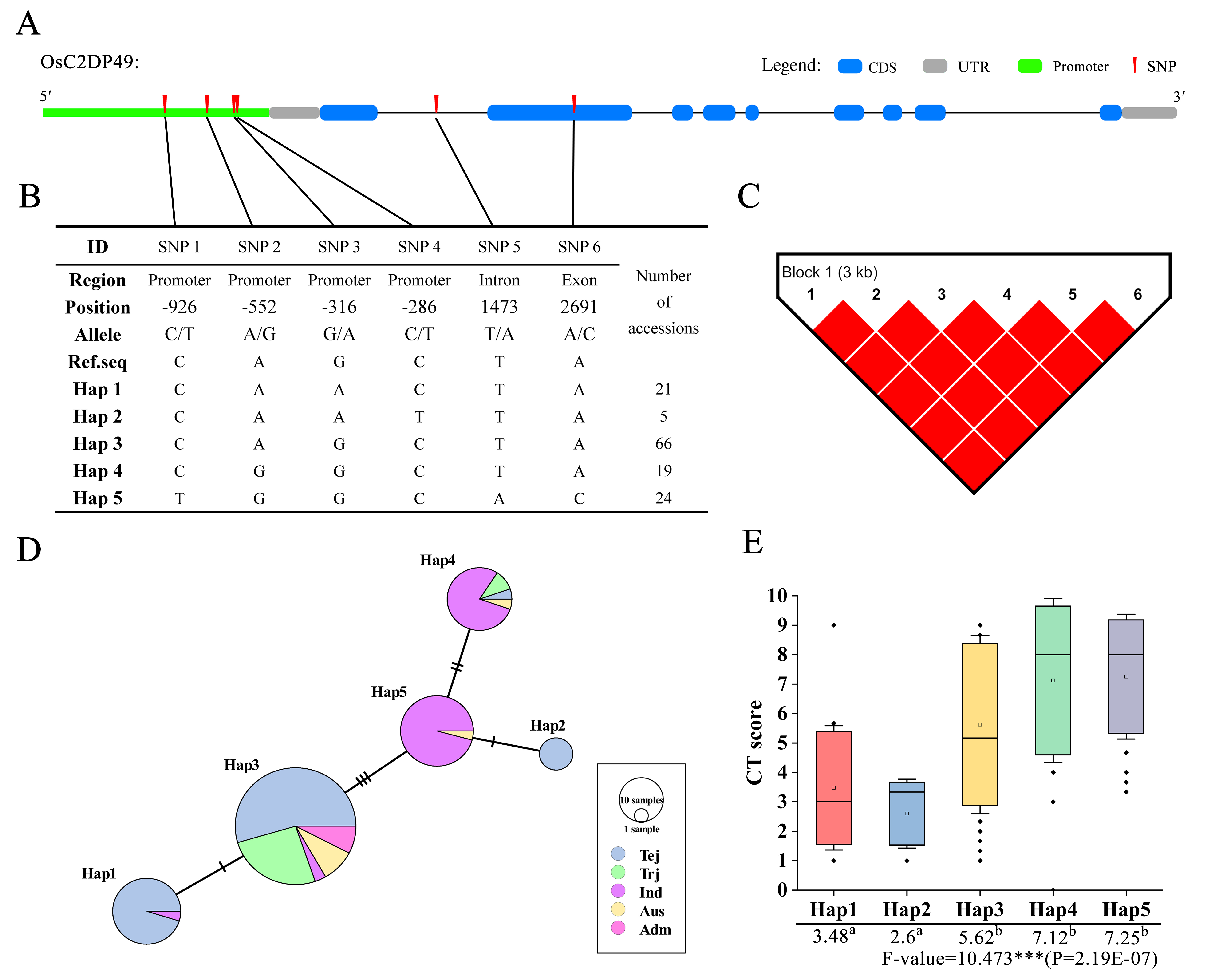

Supplement: Supplementary file 1 [file ijms-23-02221-s001.zip › ijms-1596146-supplementary/Figure S15.jpg]

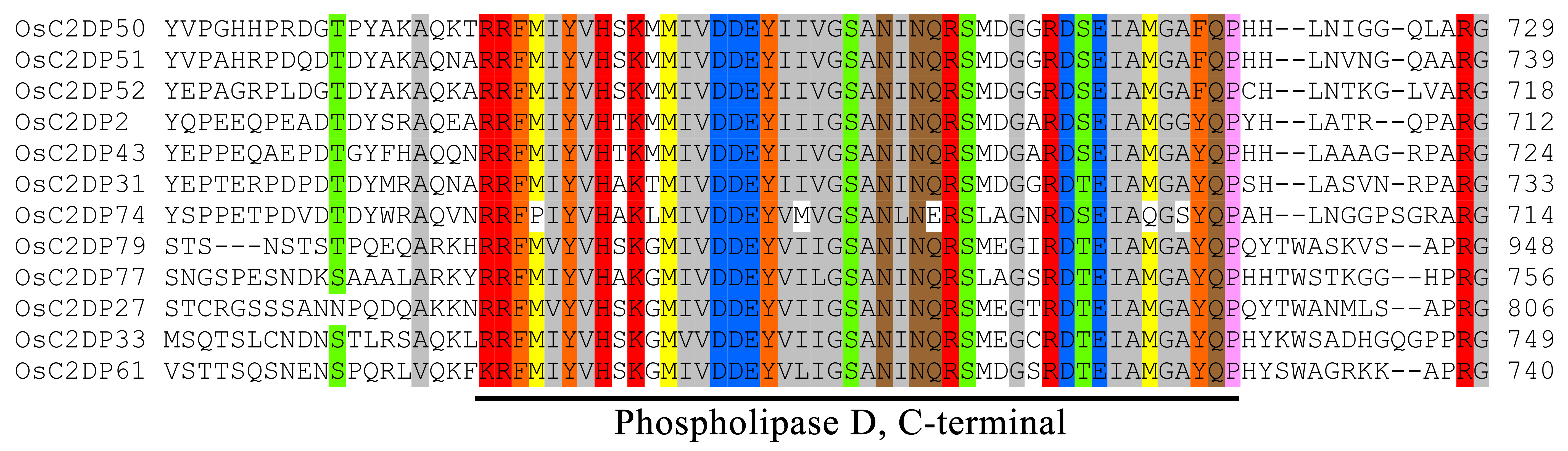

Supplement: Supplementary file 1 [file ijms-23-02221-s001.zip › ijms-1596146-supplementary/Figure S2.jpg]

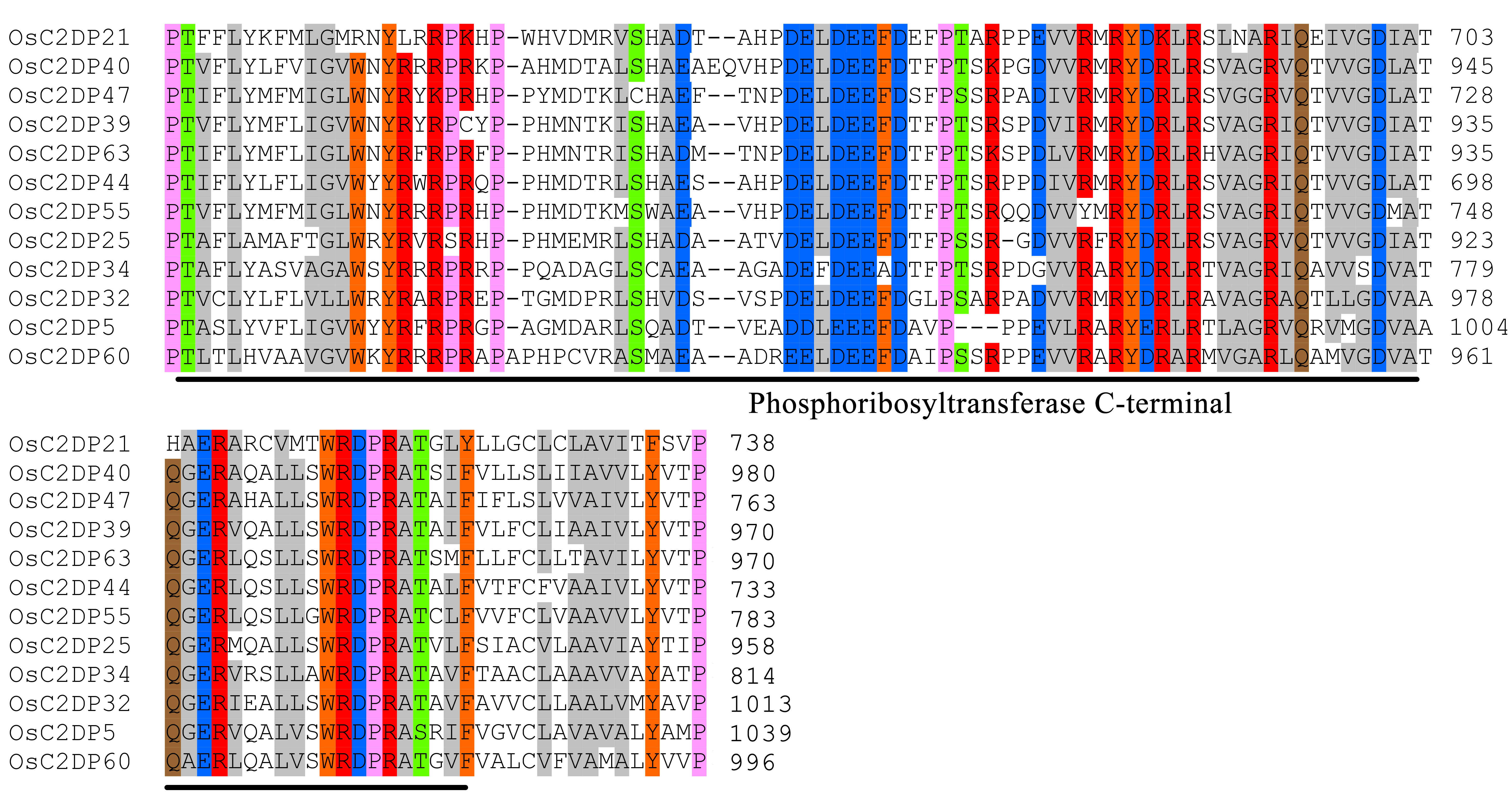

Supplement: Supplementary file 1 [file ijms-23-02221-s001.zip › ijms-1596146-supplementary/Figure S3.jpg]

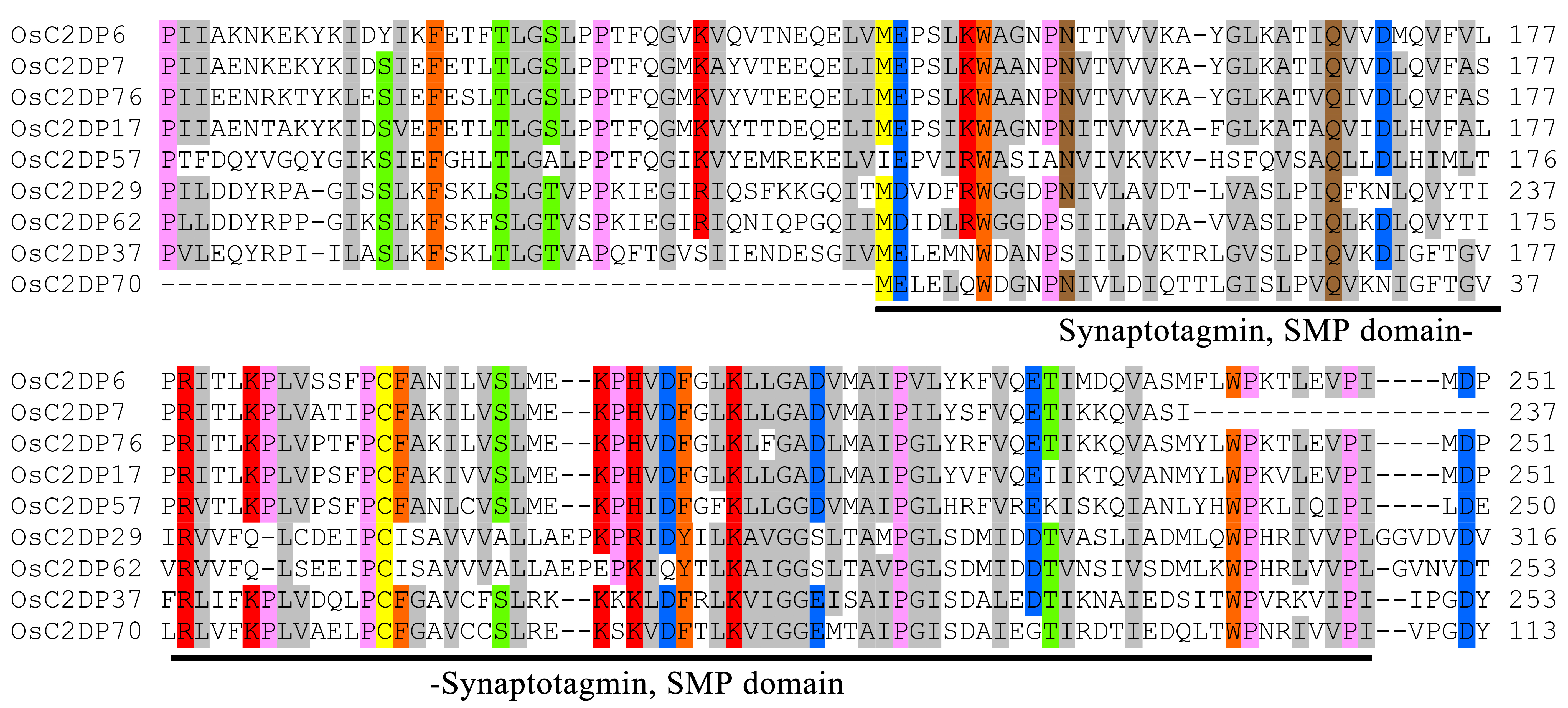

Supplement: Supplementary file 1 [file ijms-23-02221-s001.zip › ijms-1596146-supplementary/Figure S4.jpg]

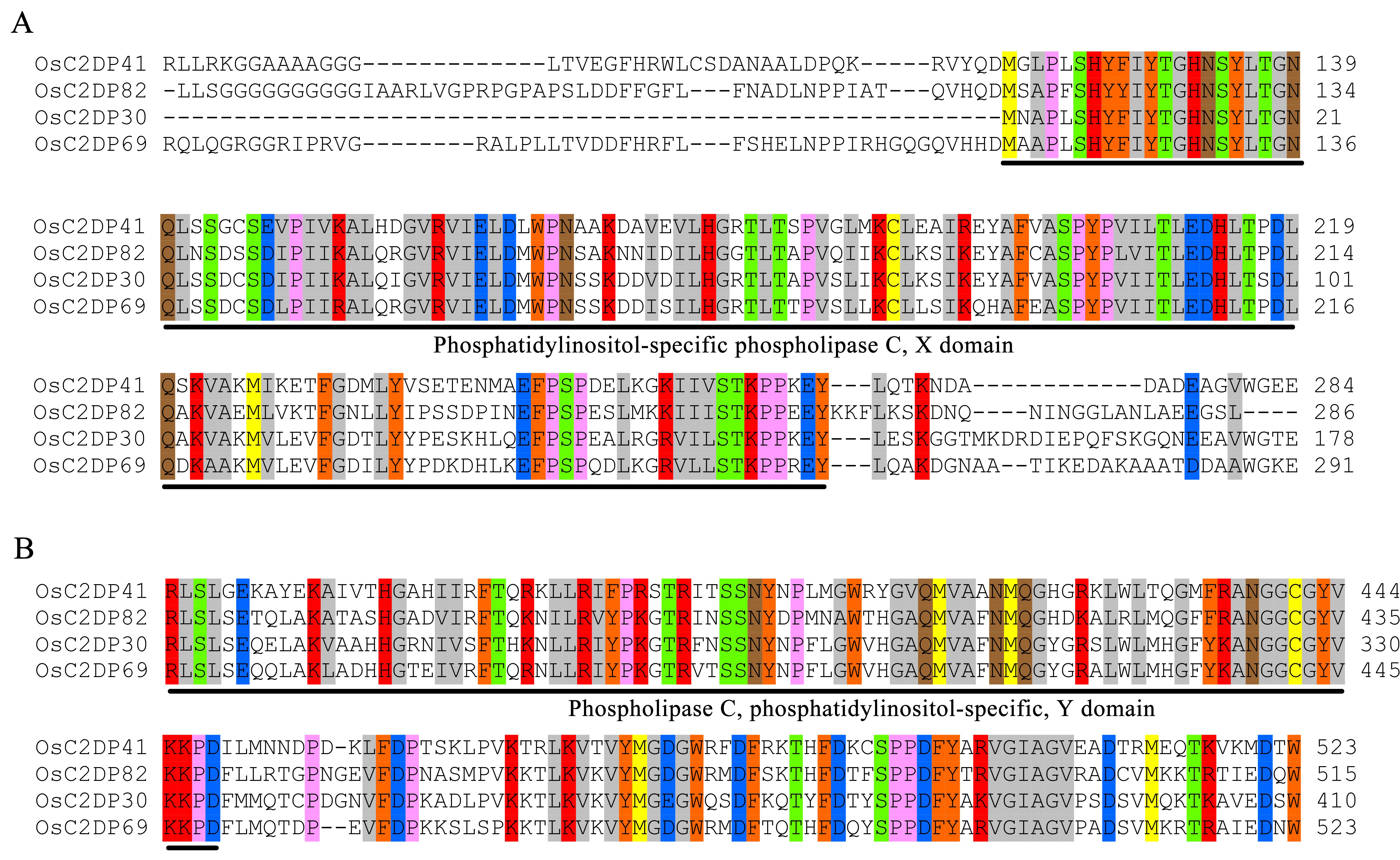

Supplement: Supplementary file 1 [file ijms-23-02221-s001.zip › ijms-1596146-supplementary/Figure S5.jpg]

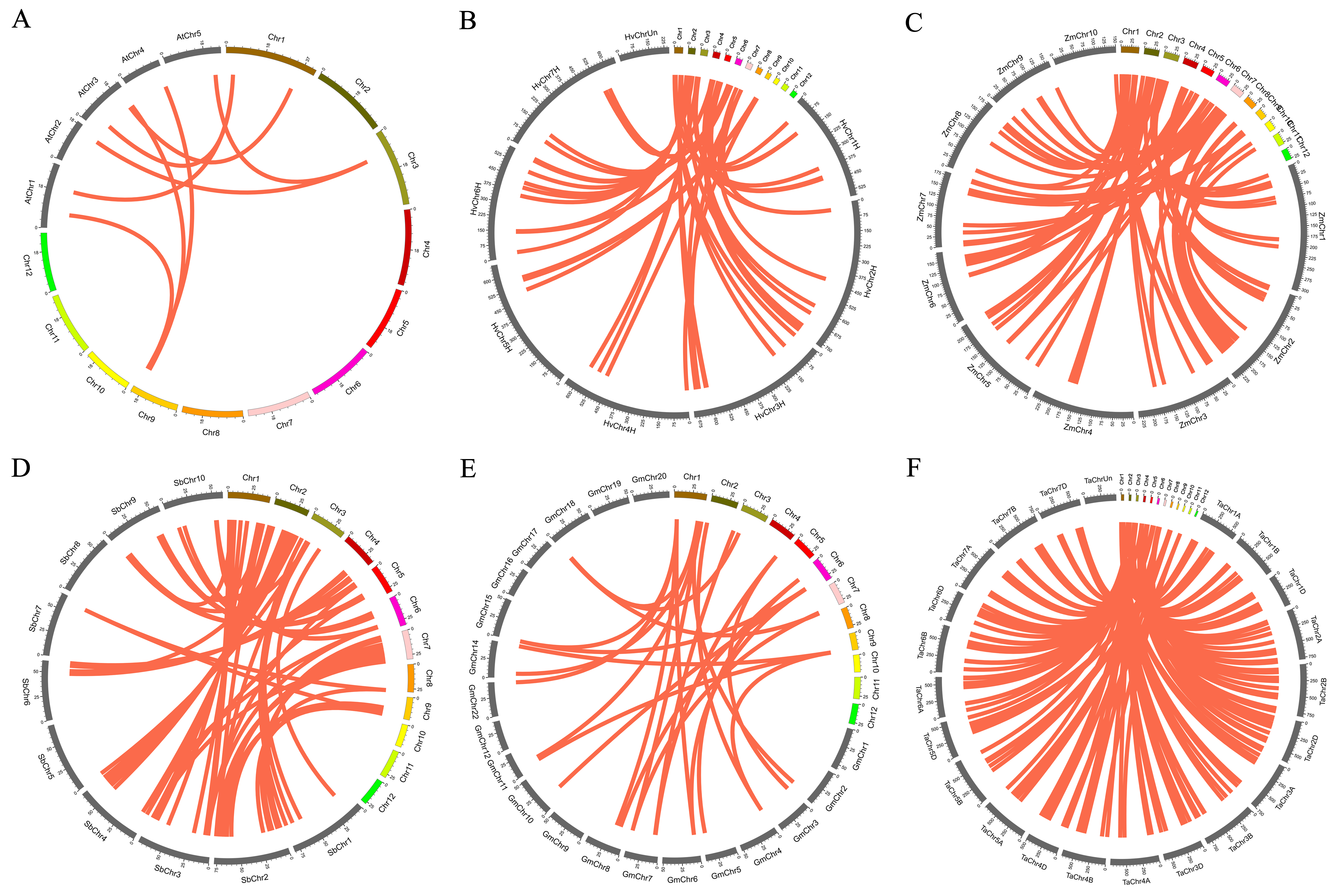

Supplement: Supplementary file 1 [file ijms-23-02221-s001.zip › ijms-1596146-supplementary/Figure S6.jpg]

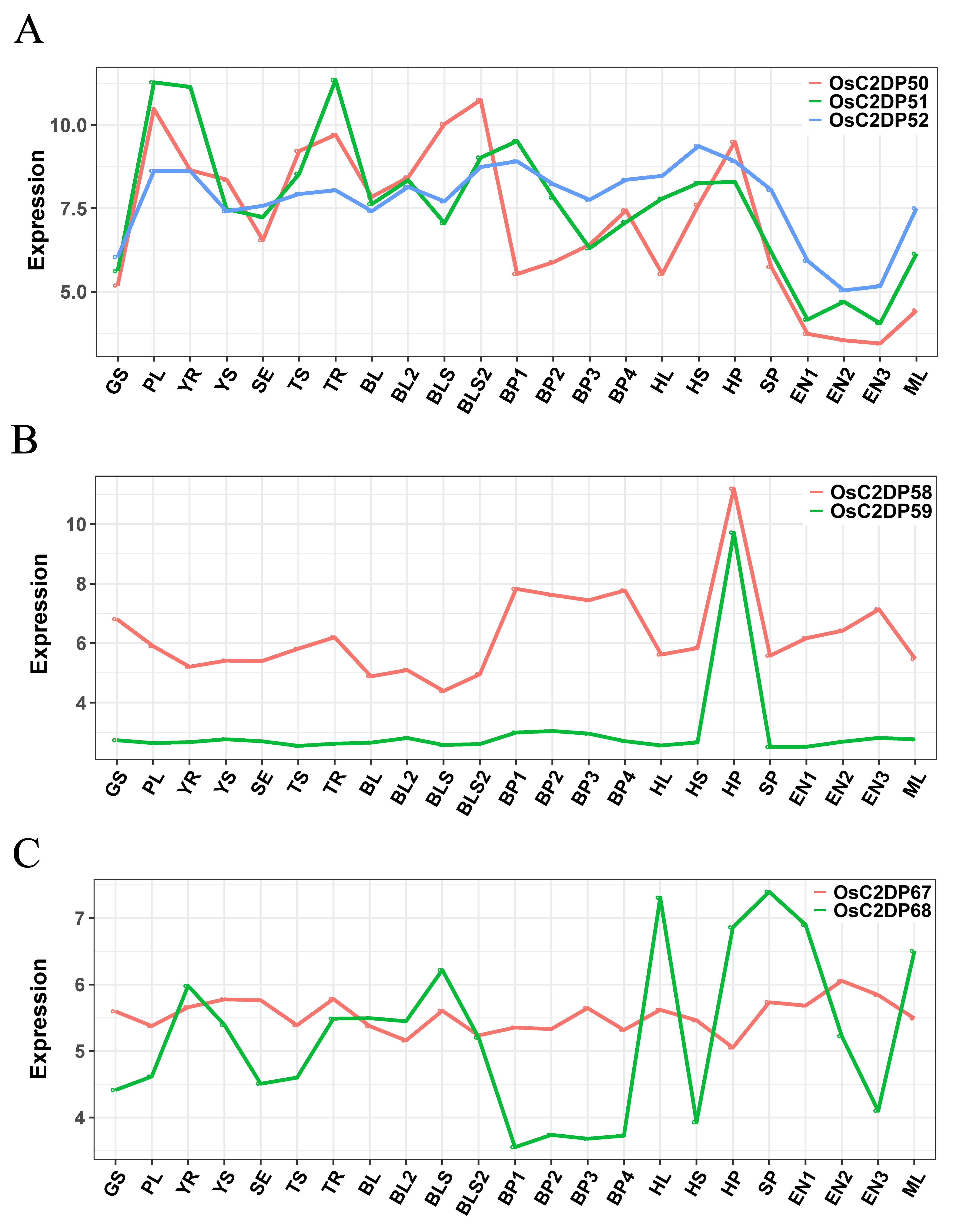

Supplement: Supplementary file 1 [file ijms-23-02221-s001.zip › ijms-1596146-supplementary/Figure S7.jpg]

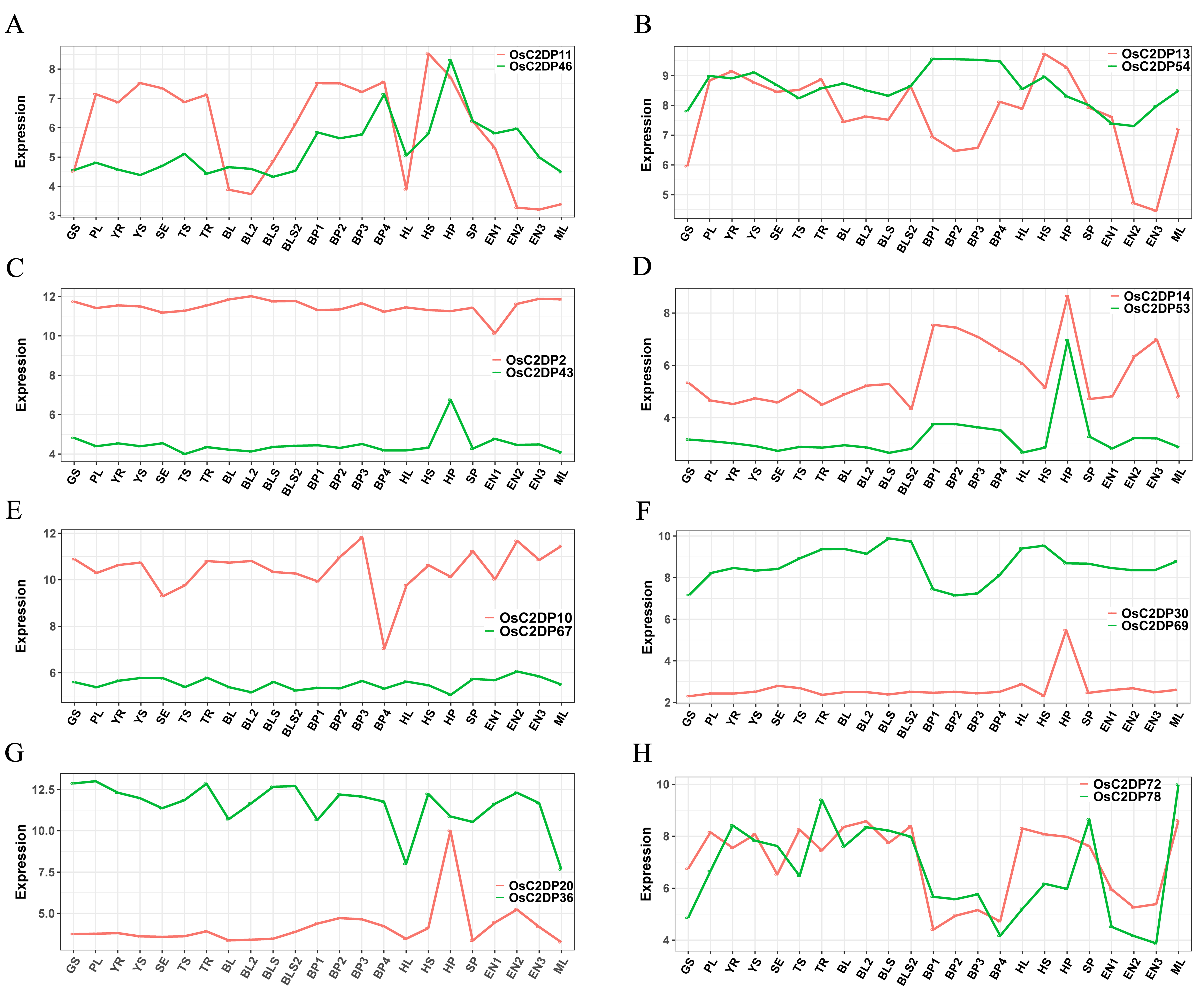

Supplement: Supplementary file 1 [file ijms-23-02221-s001.zip › ijms-1596146-supplementary/Figure S8.jpg]

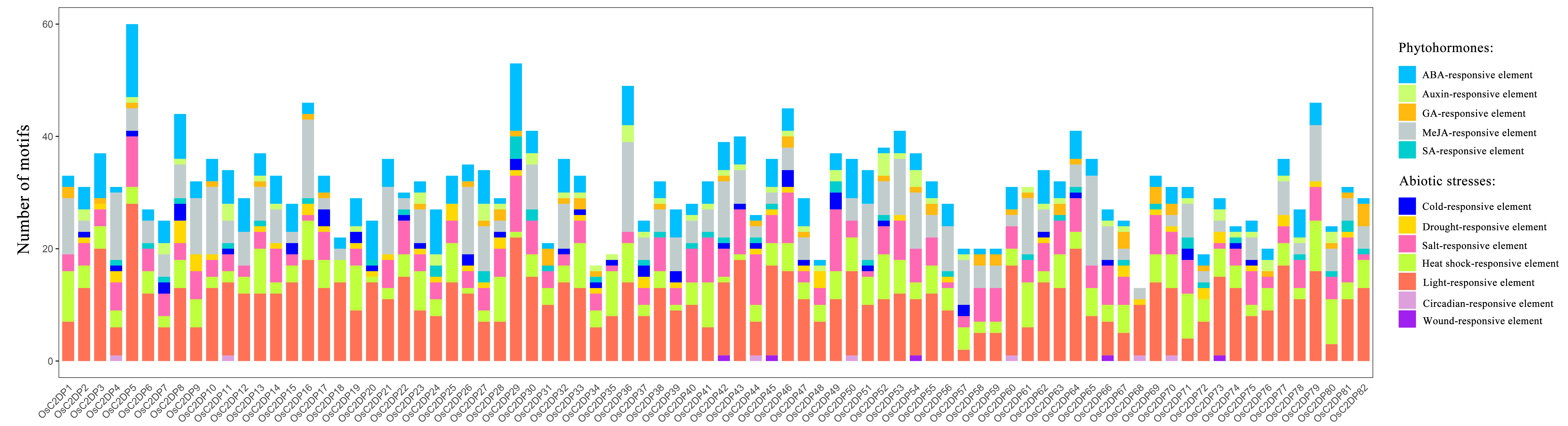

Supplement: Supplementary file 1 [file ijms-23-02221-s001.zip › ijms-1596146-supplementary/Figure S9.jpg]
